# Qualitative comparison of decalcifiers for mouse bone cryosections for subsequent biophotonic analysis

**DOI:** 10.1038/s41598-024-84330-2

**Published:** 2025-01-07

**Authors:** Shibarjun Mandal, Ramya Motganhalli Ravikumar, Astrid Tannert, Annett Urbanek, Rustam R. Guliev, Max Naumann, Sina M. Coldewey, Uta Dahmen, Lina Carvalho, Luís Bastião Silva, Ute Neugebauer

**Affiliations:** 1 Leibniz Institute of Photonic Technology (Member of Leibniz Health Technologies, Member of the Leibniz Centre for Photonics in Infection Research, LPI), 07745 Jena, Germany; 2 Center for Sepsis Control and Care, Jena University Hospital, 07747 Jena, Germany; 3 Institute of Physical Chemistry and Abbe Center of Photonics, Friedrich Schiller University Jena, 07743 Jena, Germany; 4 Department of Anesthesiology and Intensive Care Medicine, Jena University Hospital, 07747 Jena, Germany; 5 Septomics Research Center, Jena University Hospital, 07745 Jena, Germany; 6 Experimental Surgery, Clinic for General, Visceral and Vascular Surgery, Jena University Hospital, 07747 Jena, Germany; 7 Institute of Anatomical and Molecular Pathology, Faculty of Medicine, University of Coimbra, Coimbra, 3004-504 Portugal; 8 BMD Software, PCI-Creative Science Park, Ílhavo, 3830-352 Portugal

**Keywords:** Decalcification, Raman imaging, Bone, Histology staining, Immunofluorescence labelling, Confocal laser scanning microscopy, Imaging studies, Bone

## Abstract

Bone tissue, with its complex structure, often necessitates decalcification of the hard tissue for ex vivo morphological studies. The choice of a suitable decalcification method plays a crucial role in preserving desired features and ensuring compatibility with diverse imaging techniques. The search for a universal decalcification method that is suitable for a range of biophotonic analyses remains an ongoing challenge. In this study, we systematically assessed five standard bone decalcification protocols, encompassing strong mineralic acids (3% and 5% nitric acid), a commercially available formulation of hydrochloric and formic acid), as well as weak organic acids (5% trichloroacetic acid and 8% formic acid), and a chelating agent (25% ethylenediamine-tetraacetic acid) with varying decalcification durations, using mouse long bones as our experimental model. Our imaging analysis panel included classical histological staining (Hematoxylin and Eosin, H&E), immunofluorescence staining, and label-free Raman microspectroscopic imaging. We used cryosections instead of paraffin sections since paraffin interferes with tissue Raman signals. This approach is not as commonly used as it is more prone to handling artifacts, but is the preferred method for subsequent Raman analysis. Decalcification efficacy was evaluated based on various qualitative and some quantitative imaging parameters by 2–3 independent observers. Our systematic approach revealed that the chelating agent, when used for 24 h, optimally preserved bone features and, thus, would be the ideal decalcifying agent for comprehensive subsequent analysis. However, the choice of decalcifier and the ideal decalcification duration may vary depending on the type and thickness of bone, necessitating tailored adjustments to meet specific experimental requirements.

**Supplementary Information:**

The online version contains supplementary material available at 10.1038/s41598-024-84330-2.

## Introduction

Bone tissue is complex hard tissue that – as part of the skeleton – provides structure and support for the body and protection for the other soft organs of the body^1^. The ability to visualize and understand the intricate three-dimensional architecture of bone is of paramount importance for discerning the underlying causes of skeletal diseases and injuries. Thus, several histological and histochemical staining methods followed by histologic evaluation have been established, such as e.g., hematoxylin-eosin (H&E) or Gomori’s trichome staining techniques^2^. To facilitate histologic workup, especially the process of sectioning prior to staining, decalcification is often necessary. Decalcification entails the selective demineralization of hydroxyapatite, the primary mineral component of bone while preserving the delicate organic matrix^3^. The choice of decalcification method significantly impacts the preservation of tissue morphology, and antigenicity for immunostaining. Traditional decalcifying agents include strong inorganic acids (e.g., hydrochloric and nitric acid) or weaker organic acids (e.g., acetic and formic acid). These acids facilitate the breakdown of hydroxyapatite and the release of calcium ions through protonation of the phosphate groups. However, if applied too long, they can also lead to excessive demineralization, resulting in the loss of morphological features, antigen masking, and tissue damage^4,5^. Alternatively, chelating agents like ethylenediamine-tetraacetic acid (EDTA) can be employed for decalcification. These agents facilitate the removal of calcium ions from the hydroxyapatite by complexing the contained calcium ions while minimally disrupting the organic tissue matrix, thereby preserving fine structural details and antigen epitopes for optimal histological staining and immunofluorescence. Strong acids exhibit rapid decalcification action, but require careful monitoring to prevent damage to the remaining bone tissue, especially if the tissue is exposed to the acid for an extended duration. In contrast, weak acids and chelating agents have a slower action, resulting in a more delicate interaction with the sample, but requiring longer decalcification times^5^. The choice of the decalcifying agent in pathology and research laboratories considers factors such as case urgency, subtle preservation of bone marrow, size and type of bone, and subsequent analysis method (e.g. staining techniques). In recent years, comparison of different decalcifiers gained a lot of attention, in particular, if the decalcified bone should be characterized by more than one analysis method. It was found that inappropriate use of the decalcifying agent can damage the remaining tissue structure which might obscure the subsequent analysis. This is of high importance, if the subsequent work up involves immunohistochemistry analysis^6,7^ or nucleic acid analysis, such as (fluorescence) in situ hybridization^6,8,9^, DNA strand break analysis by terminal deoxy(d)-UTP nick-end labelling (TUNEL)^10^ and DNA and RNA integrity and mass analysis using polymerase chain reaction (PCR)^6,8,11^. Table 1 provides a short overview of those studies after 2017, including this study (see Supplementary Table S1 for studies published before 2017).

**Table 1:** Overview of recent (2017 and newer) studies on different decalcifiers for subsequent bone tissue characterization. Only studies using plain decalcifiers are included here. Supplementary table S1 also lists some older studies (before 2017) using plain decalcifiers. Further studies are available that use microwave, ultrasound or heating to improve the decalcification process, however, those have not been considered here. Please also note, bone thickness and width, as well as bone donor age significantly affect required decalcification times. Therefore, we have omitted times in this table. Studies are ordered according to bone origin (from human/large animal to small animal).

References	Bone used	Decalcifier used			Recommendation for best cellular quality				
		Strong acid	Weaker organic acid	Chelater	H&E	Immuno-histochemistry	Raman	Nucleic acid analysis	Combined
Cornelison et al.^31^	Human cranial bone	N (5%), H (7%)		EDTA (10%)	EDTA (if speed is relevant: N)				
Miquelestorena-Standley et al.^5^	Human bone	H	F	EDTA		EDTA (and if short F)		EDTA for in situ hybridization, For other: EDTA and F	
Pieroh et al.^44^	Human acetabular labra		TCA (6%)	EDTA (30%, Chelaplex®)	Both: TCA & EDTA	EDTA + methyl benzoate			
Pang et al.^25^	Porcine femurs	H (0.5 M), H/EDTA mixtures	F (0.5 M),	EDTA (1 M),	.		H (fast) EDTA (slow)		
Broomfield et al.^45^	Ovine shoulder and spine	TFA (8% and 20%), HF (XL-Ca)l	F (8% and 20%) Formical-4,		20% F *Also included effect of different temperature, not mentioned here				
Alaeddini et al.^46^	Rat hemimaxillae and rabbit parietal bones	N (5%, 10%)	F (5%, 10%), Gooding-Stewart liquid (= F + formalin)	EDTA	5% F EDTA *Also included effect of different fixatives, not mentioned here				
Amirtham et al.^47^	Rabbit knee joints		F (20%)	EDTA (10%)	Both comparable	EDTA (if time is not critical)			
Marinopoulos et al.^48^	Rat maxillas and mandibles	H (Decal Stat)	F (Immunocal)		F (Immunocal)	Both, comparable with different decalcification times			
Liu et al.^29^	Rat femurs	N (3% & 5%) HF (8%)		EDTA (10%)	3% N	EDTA, 5% N			
Freitas et al.^49^	Joints from mice and rats	N (10%),		EDTA (12.5%)			EDTA		
Savi et al. ^4^	Rat mandibles	N (5%)	F (10%)	EDTA (10%)	EDTA (N if speed is crucial)	EDTA			
Bogoevski et al.^50^	Mouse and Rat Tibiae	H (5%) N (5%)	F (10%)	EDTA (10%)	EDTA	EDTA			
This study	Mouse tibia, humerus, femur, ulna	N (3%, 5%) HF	F (8%) TCA (5%)	EDTA (25%)	F, N	EDTA	EDTA		EDTA

EDTA: Ethylenediamine-tetra acetic acid; N: Nitric acid; F: Formic acid; HF: Hydrochloric and formic acid, H: Hydrochloric acid; TFA: Trifluroacetic acid; TCA: Trichloroacetic acid.

A relatively new method that can provide molecular imaging in a label-free manner from a biological tissue sample is Raman spectroscopy. In recent years, it was successfully applied to characterize bone tissue, e.g. to provide insights into collagen secondary structure^12^ or matrix quality^13^, study bone-implant interfaces^14^, follow bone changes in the presence of bacteria^15^ or during bone repair^16^, reveal differences between diseased tissue and healthy bone^17^, differentiate osteoarthritis bones according to age and sex^18^ or even provide insights from a bone from a prehistoric ancestor^19^. Raman spectra also showed a significant difference between the different entities. Most studies involving Raman spectroscopic characterization of bone were focusing on calcified bone. However, if Raman analysis is supposed to be done in parallel to histochemical analysis, a similar workflow should be adopted which also involves decalcification. No systematic study was performed to compare the effect of different decalcifiers for subsequent biophotonic analysis, such as classical microscopic analysis after histological staining, immunofluorescence imaging as well as label-free Raman imaging.

In this article, we present a semi-quantitative comparison of different existing standard bone decalcification protocols, with the aim to identify the most suitable protocol for subsequent multimodal imaging covering classical histological staining (H&E), immunofluorescence staining and imaging and label-free Raman microspectroscopic imaging of mouse bones. We chose five different decalcifying agents for our study. These included strong acids such as nitric acid (in two different concentrations), and a combination of hydrochloric and formic acid. We also used weak acids like trichloroacetic acid and formic acid, along with the chelating agent EDTA. Following decalcification, we used bone cryosections instead of paraffin sections to avoid the strong Raman signal from paraffin that would overlap with the tissue’s signal of interest^20^ and require complex spectral processing to remove^21^. Experiments were conducted in triplicates for each decalcifying agent, with decalcification durations of 2, 6, 12, 24, 48, and 72 h, depending on the agent used. Decalcification performance was evaluated based on images from different imaging modalities and assessed by 2–3 independent observers. Although cryosections introduced handling artifacts, particularly during immunofluorescence staining, we selected the best samples for evaluation. By employing this systematic approach, we identified the most suitable decalcifier for effective multimodal imaging applications with mouse bones. However, we acknowledge that the optimal decalcifier or protocol may need adjustments for larger or different types of bones.

## Results and discussion

Detailed assessments of the decalcification protocols, tailored to each imaging modality, are provided in the following sections. The scoring scheme, both quantitative and qualitative, used for result evaluation is illustrated in Supplementary Figure S1. The individual scores from each observer for the three different batches can be found in the Supplementary Evaluation Excel file, while in the main manuscript averaged values are presented.

### Assessment of decalcification by Raman spectroscopic characterization of the compact bone

**Fig. 1:**
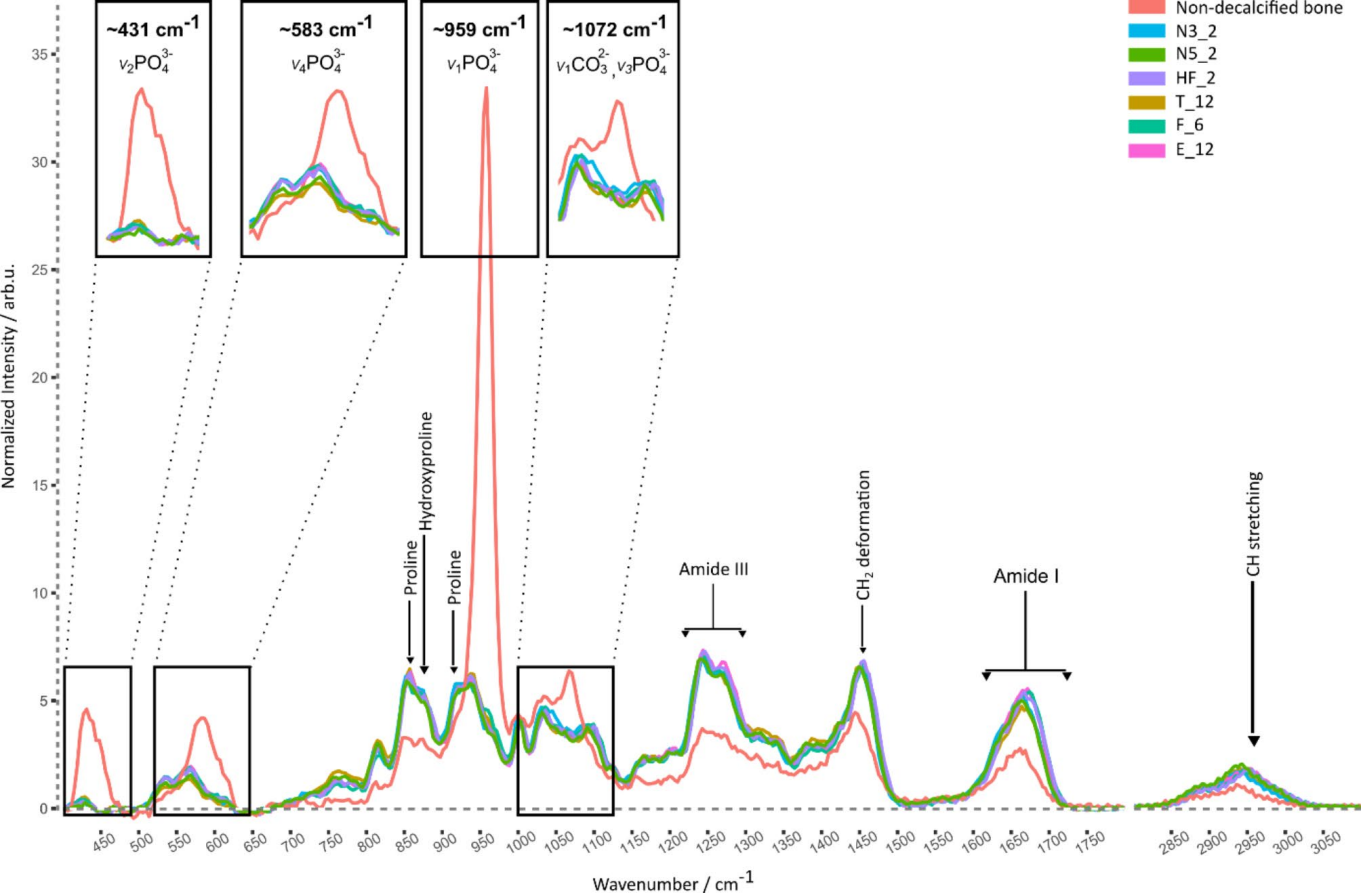
Baseline-corrected Raman spectra of compact bone after decalcification with different decalcifiers using the shortest investigated decalicification duration (cyan: 3% nitric acid for 2 h (N3_2), dark green: 5% nitric acid for 2 h (N5_2), violet: hydrochloric and formic acid for 2 h (HF_2), dark yellow: trichloroacetic acid for 12 h (T_12), green: formic acid for 6 h (F_6), pink: EDTA for 12 h (E_12)) in comparison to non-decalcified (original) bone (red). Insets show magnified phosphate bands around 431, 583, 959 and 1071 cm^− 1^, while arrows indicate collagen bands. It can be seen that for all decalcifiers, the calcium phosphate bands are missing indicating complete decalcification already at the first decalcification time point, in particular at two hours for nitric acid as well as for hydrochloric and formic acid, at six hours for formic acid and at twelve hours for EDTA as well as for trichloroacetic acid.

One exemplary spectrum for each decalcifier with the shortest decalcification time acquired from the compact bone tissue is presented in Fig. 1, together with a Raman spectrum of non-decalcified compact bone tissue. The spectrum of the calcified bone shows a dominating Raman band around 959 cm⁻¹, and further prominent Raman bands around 431 cm⁻¹, 583 cm⁻¹, and 1072 cm⁻¹. All those four bands can be assigned to vibrations of the phosphate moiety ($\:P{O}_{4}^{3-}$) of the hydroxyapatite, the mineral component of the bone. The band around 1072 cm^− 1^ also contains in addition some contributions from carbonate ($\:C{O}_{3}^{2-}$)^15,22^. Phosphate bands were exclusively observed in the spectrum of the calcified bone, but in none of the decalcified bone spectra as is clearly visible also in the inset of Fig. 1. This indicates that decalcification is complete and all mineral components have been removed already after the minimal incubation period recommended for the given reagent.

Raman spectra of decalcified bone tissues are dominated by spectral contributions of the collagen matrix (Fig. 1). Typical spectral contributions are found around 921 and 855 cm^−1^ (both assigned to proline), 876 cm^−1^ (hydroxyproline), a broad band around 1243–1320 cm^−1^ (amide III) and around 1616–1710 cm^−1^ (amide I), as well as the CH_2_ deformations around 1450 cm^−1^.^23^ Spectra presented in this study are in line with previously analyzed Raman spectra of decalcified bone tissue^24,25^. Comparing the compact bone spectra obtained after decalcification with different decalcifiers shows high similarities between all spectra, suggesting that decalcification has minimal impact on collagen, preserving its structural information. The quality of Raman spectra of compact bone was similar for all decalcification protocols yielding consistently high signal-to-noise ratio (SNR) (see below).

### Assessment of hematoxylin and eosin (H&E) staining after decalcification

**Fig. 2:**
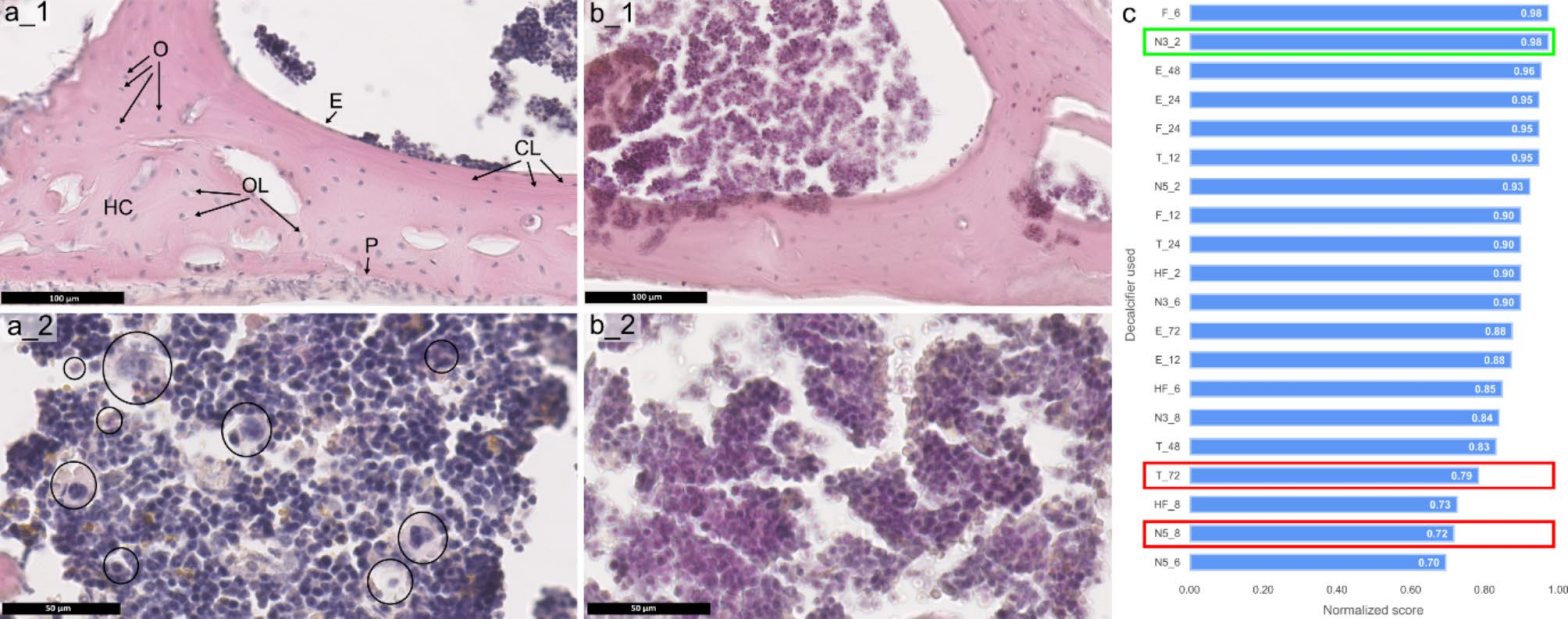
Comparative analysis of H&E stained bone tissue images for different decalcification protocols. (**a_1**) Decalcification protocol N3_2. Detailed view of compact bone after decalcification with 3% nitric acid for 2 h exhibiting relevant bone features, including osteocytes (O), osteocytes within lacunae (OL), cement line (CL), Haversian canal (HC), endosteum (e), and periosteum (P). (**a_2**) Bone marrow region from the same decalcification protocol (N3_2), predominantly comprising myeloblasts or common progenitor cells or erythrocytes, with a few other cells also visible, that could be megakaryocytes, mast cells or monocytes/macrophages (highlighted in a circle). (**b_1**) Decalcification protocol: T_72. Image illustrating visible cement lines, while osteocytes appear inadequately stained and, thus, less distinguishable. (**b_2**) Decalcification protocol: N5_8. Image of bone marrow displaying clustered cell nuclei with limited distinguishability, accompanied by unclear distinction between hematoxylin and eosin. (**c**) Evaluation chart summarizing normalized scores after H&E staining following different decalcification methods and durations of decalcification. The decalcifier used for panel (**a**) is highlighted in green, while those used in panel (**b**) are highlighted in red. Abbreviations of decalcifiers are found in Table 2.

A clear visualization of the relevant bone features obtained after H&E staining concerning compact bone tissue and bone marrow section is depicted in Fig. 2a. In the compact bone tissue (Fig. 2a_1), cement lines can be clearly recognized and osteocytes are visible within the lacunae. Moreover, the distinct Harversian canal structures can be observed. Cells adjacent to the periosteum and endosteum could be osteoblasts and osteoclasts. Overall, observed cellular and structural features are visualized as expected for healthy and mature bone tissue used in our study ^26,27^.

Figure 2a_2 depicts a well-stained bone marrow section. The nuclei and cytoplasm of the cells exhibit clear and distinguishable staining intensity throughout the section. In the depicted region, many cells with a large nucleus are visible, as is expected for bone marrow. According to the morphological features, the cells belong to the hematological precursor cells and could be hemocytoblasts or common progenitor cells, or erythroblasts. Additionally, there are other cells present (indicated by the black circle), which could be adipocytes, megakaryocytes, or myeloid cells. For an in-depth further differentiation, further analysis would be needed.

Both images in Fig. 2a, showing high quality H&E staining, originated from the bone after decalcification with 3% nitric acid for 2 h (N3_2). Other decalcifying protocols that led to similar desirable outcomes within a short period of time included the use of EDTA, formic acid, as well as trichloroacetic acid, specifically for the shortest time point (T_12). This can be observed in the summary of the evaluation chart in Fig. 2c (decalcifiers obtaining the highest scores). This means, decalcifying protocols based on weaker organic acids, chelators, as well as strong inorganic acids in lower concentrations and short decalcification times can be suitable to obtain bone sections which yield high contrast images after H&E staining. For longer decalcification periods, chelating agents are more appropriate. All of those decalcification protocols have also been extensively documented in the literature (also please refer to the Table 1), demonstrating their effectiveness in efficiently removing calcium from bone tissue and also for being suitable for preserving tissue integrity for subsequent H&E staining^5,28,29^.

On the other hand, Fig. 2b showcases some undesirable features observed after decalcification with other protocols resulting in low scores. As depicted in Fig. 2b_1, decalcification with trichloroacetic acid for 72 h (T_72), resulted in inadequate staining of osteocyte nuclei in the compact bone region leading to poor visibility of those cells. Figure 2b_2 shows a bone marrow section with poor discernibility of individual cells and nuclei after decalcification with 5% nitric acid for 8 h (N5_8). As shown in the summarizing evaluation chart in Fig. 2c, the lowest scores indicative of the highest damage were obtained for long decalcification times with strong acid-based decalcifiers. Remember, that it was shown in Fig. 1 that the shortest decalcification times for each decalcifier already led to full decalcification. Thus, decalcifiers based on strong inorganic acids, which very rapidly remove the calcium minerals, have started to macerate tissue after longer decalcification times, which resulted in impaired staining, especially of basophilic elements, such as cell nuclei^3,30^. Thus, type, concentration and timing of the used decalcifier play an important role to achieve optimal staining results. H&E tissue images of all used decalcifiers and time points are presented in Supplementary Figure S2.

### Assessment of fluorescence staining after decalcification

**Fig. 3:**
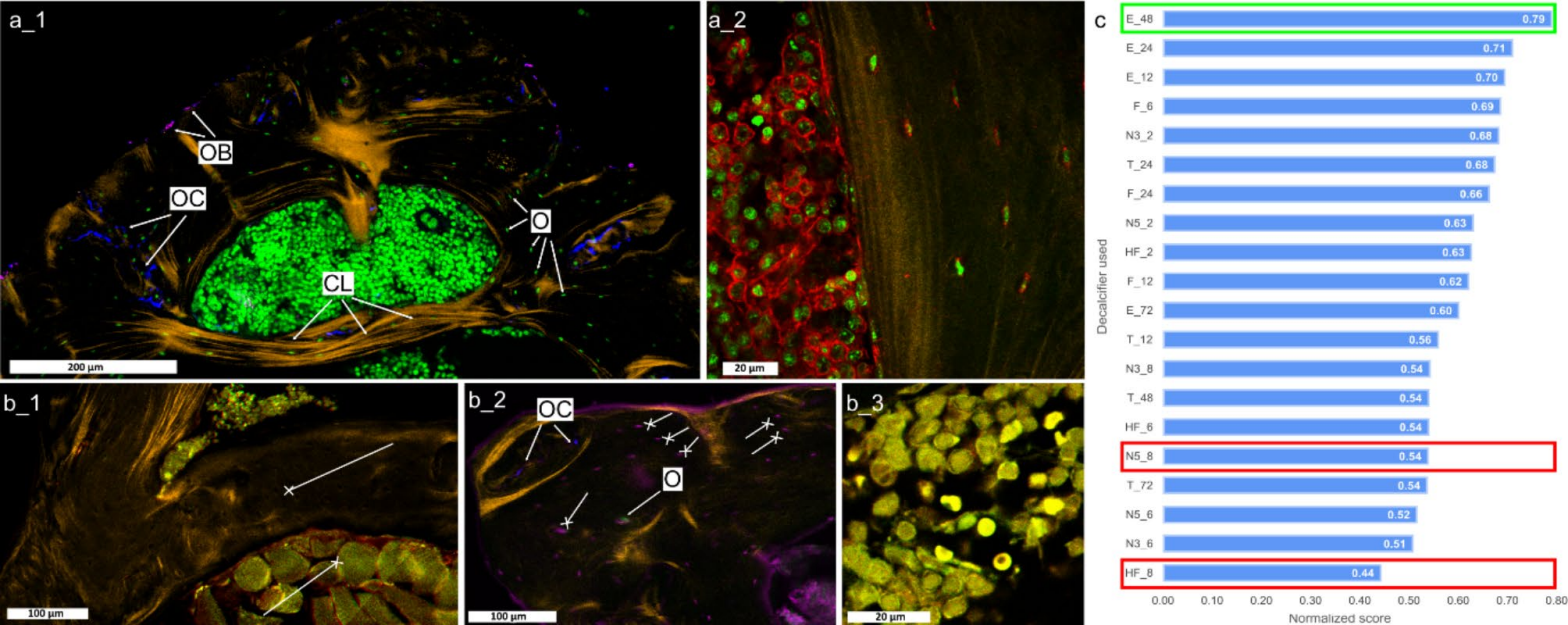
Comparative analysis of fluorescence-stained bone tissue images and evaluation chart displaying normalized scores rated by observers for various decalcification protocols. (**a_1**) Decalcification protocol: E_48. Detailed view of compact bone regions exhibiting relevant bone features, including osteocytes (O), traces of osteoclasts (OC), and osteoblasts (OB, pink), along with the collagen lamella (CL, yellow). (**a_2**) Bone marrow region displaying clearly visible cell nuclei (green). Red staining shows actin. (**b_1**) Decalcification protocol: N5_8. Nucleic acid stain Sytox-green (green/yellow) also unspecifically stains muscle tissue (cross-head in lower half of the figure) while osteocytes in compact bone are not stained (cross-head in the middle of the figure). (**b_2**) Decalcification protocol: HF_8. Traces of osteoclasts (OC) are evident (arrow head, OC), but the pre-osteoblast stain dominates the tissue or the nuclei-specific stain is not sufficiently absorbed, resulting in overlapping and non-specific staining, making the appearance of osteocytes resemble osteoblasts (cross-head). Only a few regions show specific staining of nuclei (arrow-head, O). (**b_3**) Probable loss of bone marrow definition due to the decalcification protocol, also non-specific staining causing actin (red) and nucleic acid stain (green) to overlap resulting in yellow staining. (**c**) Evaluation chart summarizing normalized scores for fluorescence staining after decalcification with different decalcifiers and durations of decalyifying procedure. The decalcifier used for panel (**a**) is highlighted in green, while those used in panel (**b**) are highlighted in red. Abbreviations of decalcifiers are found in Table 2.

The immunofluorescence-stained bone section after decalcification with EDTA for 48 h (E_48) revealed clear, distinct, and specific staining of the respective molecular structures, as shown in Fig. 3a_1. Specifically, in the compact bone, the nuclei and actin cytoskeleton of osteocytes are easily distinguishable. Additionally, traces of osteoblasts and osteoclasts can be identified within the compact bone tissue. Collagen fibers were visualized using label-free second harmonic generation (SHG) microscopy. Notably, collagen was easily detectable with comparable quality in all investigated tissue slices from all used decalcifiers and thus, this parameter was not included in the final score. Figure 3a_2 demonstrates successful staining and imaging of the bone marrow cells without any disruption or interference. By utilizing a high-resolution scan using a 63 x objective we were able to clearly discriminate and identify individual cells. Based on nuclei morphology, these cells are most likely myeloblasts and erythroblasts, as is expected from a typical bone marrow in long bones, and were already visualized in the H&E images (Fig. 2b_1).

In contrast, Fig. 3b_1 shows a fluorescence image of a stained bone section which exhibits several shortcomings. These include unintended staining of the muscle tissue by the nuclear stain Sytox green while staining of osteocyte nuclei within the compact bone is poor. Thus, characteristic features of the bone tissue are not fully visualized.

Figure 3b_2 shows another sample of poor fluorescence staining after decalcification with 5% nitric acid for 8 h (N5_8). Specific staining of selected relevant antigens is lost and an overlapping staining is observed for osteoblasts and osteocytes. Consequently, the reliable identification and assessment of osteocytes within the tissue is hampered as only a few properly stained osteocytes are visible. Moreover, loss of specific staining is also observed for the bone marrow, resulting in loss of distinct visualization of nuclear shapes (Fig. 3b_3). This loss may be due to acids potentially damaging epitopes by hydrolyzing peptide bonds, cross-linking proteins, or causing conformational changes that reduce antibody recognition. Furthermore, after decalcification with almost all decalcifiers, except for EDTA, we obtained a rather low and non-specific signal when staining the actin cytoskeleton with phalloidin. This is visualized by comparing good actin cytoskeleton staining after decalcification with EDTA in Fig. 3a_2 and poor staining after decalcification with 5% nitric acid in Fig. 3b_3. The reason for the poor phalloidin-actin staining after decalcification with strong or weak acids might either be a disassembly of the actin cytoskeleton by the decalcification process or an impairment of the phalloidin binding site. To determine the exact mechanism preventing actin visualization by phalloidin was beyond the scope of this study. Further fluorescence images of stained bone sections from all used decalcifiers and time points are presented in Supplementary Figure S3.

To quantitatively assess the brightness of immunofluorescence staining after decalcification we defined a signal intensity index taking into account the used laser power and detector gain for the individual channels during imaging. These parameters were adjusted individually for each slice to give optimal image quality while using the detectors dynamic range (see Material and methods section for further details). The sum of the signal intensity index was highest for the protocols using 24–48 h of decalcification with EDTA (see Supplementary Figure S4). This agrees with our qualitative scoring results, especially for the channels representing actin and nuclear staining (see above).

Figure 3c summarizes the total immunofluorescence scores from all decalcifying protocols and incubation times in an evaluation chart. Decalcification with EDTA for 48 h yielded best results for subsequent (immuno)fluorescence staining and imaging. Decalcification with EDTA for 24 h and 12 h were scored as second best as shown in the score list, indicating the suitability and superiority of this gentle chelating decalcifier in preserving the integrity of cellular and extracellular components for subsequent immunohistochemistry. Our observation is in line with the findings of others, see Table 1. We confirmed that decalcification with EDTA-based protocols resulted in the preservation of tissue morphology and a high contrast staining quality enabling the reliable analysis of the tissue components using (immuno-) fluorescence imaging (see Table 1 and Supplementary Table S1 and studies cited therein). We also confirmed that other decalcifying protocols based on strong acids have also previously been reported to cause loss of antigenicity and morphological integrity, especially after prolonged exposure (after decalcification was achieved) (see Table 1 and Supplementary Table S1 and studies cited therein).

### Assessment of Raman spectral characterization after decalcification

As mentioned above, no extensive differences in Raman spectral quality were detected in the compact bone sections after decalcification. Therefore, Raman spectral quality of the compact bone was only considered as one parameter derived from the quantification of the signal-to-noise ratio (SNR) within the final scoring (Supplementary Figure S1c). Since the SNR was sufficiently high in all Raman images (see Supplementary Figure S5a), the scores ranged from moderate to high and remained consistent across different batches (also shown in Supplementary Excel file, Sheet Raman SNR). Decalcification protocol with hydrochloric and formic acid for 6 h (HF_6), trichloroacetic acid for 12, 24 and 48 h (T_12, 24, 48), and EDTA for 12, 24 and 72 h (E_12, 24, 72) have consistently high scores across batches, indicating good SNR. No low scores were found, indicating that the specific spectral bands of decalcified bone tissue (mentioned before) were effectively detected in the compact bone.

**Fig. 4:**
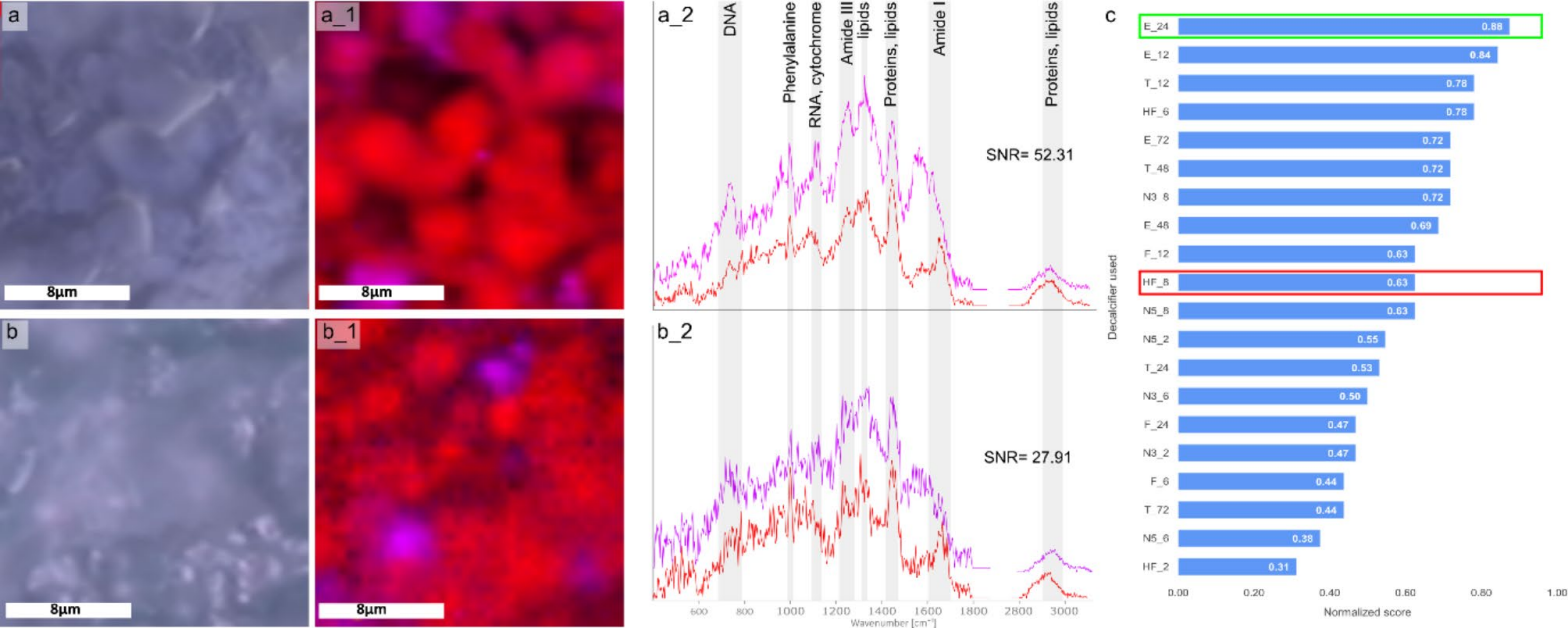
Comparative analysis of Raman measurements, image scans, and spectra of bone tissue, along with an evaluation chart displaying normalized scores for different decalcification protocols. (**a**,** b**) Bright field image of the ROI where Raman measurements were conducted. (**a**) Decalcification protocol E_24 (**a_1**) False color Raman image generated after spectra analysis, revealing nuclei-like structures. (**a_2**) EM spectra obtained from the image scan, exhibiting distinct peaks indicative of cellular features (highlighted in the figure). (**b**) Decalcification protocol: HF_8. (**b_1**) False color Raman image obtained after spectra analysis, lacking specific discernible structures. (**b_2**) EM spectra obtained from the image scan, displaying noisy spectra with only a few regions showing distinct peaks. (**c**) Evaluation chart summarizing normalized scores for Raman spectra and imaging after decalcification with different decalcifiers and durations of decalcifying procedure. The decalcifier used for panel (**a**) is highlighted in green, while those used in panel (**b**) are highlighted in red. Abbreviations of decalcifiers are found in Table 2.

Since all Raman spectra of compact bone after the different decalcification methods were of good to excellent quality, the main focus was set on characterizing spectral quality, including signal-to-noise ratio and spectral features, as well as the quality of the false color Raman images in the bone marrow sections. Figure 4 contains the evaluation chart, which demonstrates the performance of various decalcification protocols, highlighting that EDTA for 24 h (E_24) performed best (Fig. 4a). Spectral unmixing using N-FINDR algorithm yielded at least two endmembers with typical Raman bands representing the chemical composition of cellular material. Characteristic spectral features are labelled in Fig. 4a_2 and assigned in Supplementary Table S2. Clear spectral contributions from proteins are visible, e.g., with the phenylalanine band around 1003 cm^− 1^ or the amide I band around 1660 cm^− 1^. The Raman band around 1450 cm^− 1^ can be assigned to CH-deformation vibrations and could originate from lipids, proteins or carbohydrates. The endmember rich in nucleic acid contributions (Raman band around 780 cm^− 1^) is used to visualize cellular nuclei in Raman false-color images. In the image from a bone marrow section depicted in Fig. 4a_1, lobulated nuclei like structure, possibly resembling megacaryotes, are clearly visible. These findings indicate that the decalcification protocol used in our study successfully preserved essential cellular information, ensuring its suitability for future analysis and investigations.

When comparing to other decalcification protocols, such as to decalcification with strong inorganic acids with long decalcification times, e.g. the one shown in Fig. 4b (decalcification protocol: HF_8), we noticed that the essential bands are still present in the endmember spectra (Fig. 4b_2), but with higher levels of noise. This indicates that additional preprocessing steps are required to enhance the quality of the spectra for further analysis. The impact of the poor spectral quality is also evident in the Raman false color image (Fig. 4b_1), where distinct cellular shapes cannot be easily discerned. Moreover, the SNR was lower compared to the protocol with EDTA (E_24, Fig. 4a), suggesting a reduction in spectral quality with the HF_8 decalcification protocol. As discussed before, decalcification with strong acids can degrade or modify biochemical components in bone, such as proteins, lipids, and nucleic acids, which also alters the respective Raman signature. For bone marrow, the scores varied more than for the compact bone. Best performance was achieved by EDTA-based decalcification and with 12 h of trichloracetic acid or 6 h of hydrochloric acid/formic acid formulation (Fig. 4c). Raman false color images of all used decalcifiers with their respective SNR value and time points are presented in Supplementary Figure S6. The quantitative evaluation of the SNR of bone marrow-derived Raman spectra for the different decalcification procedures are given in Supplementary Figure S5b. These data agree with the qualitative scores.

In conclusion, the chelator-based protocols, such as EDTA for 24 h and 12 h (E_24 and E_12) demonstrated superior performance in terms of preserving spectral quality and enabling clear visualization of cellular structures. Other protocols, especially strong inorganic acids, such as HF_8, exhibited higher levels of noise and reduced spectral quality, making further analysis and interpretation more challenging. These findings emphasize the importance of selecting an appropriate decalcification protocol for optimal results in Raman microspectroscopy and imaging of bone samples.

### Decalcifier assessment: variations, artifacts, recommendations

**Fig. 5:**
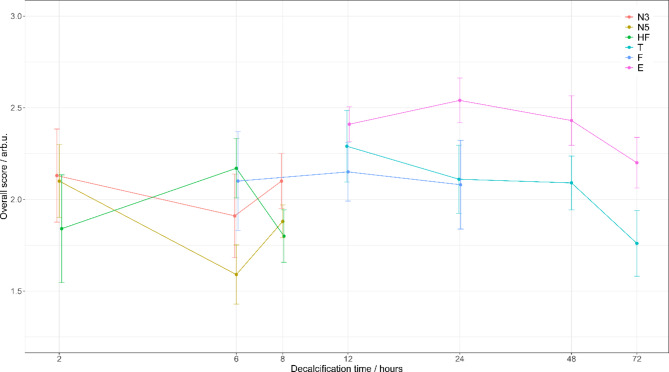
Visualization of decalcification durations and overall bone quality preservation scores obtained using sub-scores from all imaging methods. The error bars represent the standard deviation between mentioned imaging methods. Used decalifiers: N3: 3% Nitric acid, N5: 5% Nitric acid, HF: Hydrochloric and Formic acid, T: Trichloroacetic acid, F: Formic acid, E: EDTA. Decalcification time is depicted on the x-axis in logarithmic scale.

Analyzing quantitative data and qualitative scores from the observers revealed that the chelating agent decalcifier, specifically EDTA, is the most effective choice if subsequently different imaging methods should be performed using the same bone sample. Figure 5 summarizes the overall scores from the different imaging modalities for the different decalcifying protocols. Small variation of the incubations time resulted only in minor variations of the score (within standard deviation of the scores). Nevertheless, an association between decalcification duration and scores is visible. If the bone is exposed too long to the decalcification agent, the suitability of the decalcified bone specimens for subsequent photonic analysis could suffer as indicated by the lower score value for longer decalcification times in Fig. 5. This in agreement with current state of the art knowledge: initially, decalcifiers just remove the mineral part of the bone, however, if the bone is already fully decalcified, the decalcifying agent can also damage the remaining organic part. Thus, it is of utmost importance to always choose appropriate decalcification durations in preserving bone sample integrity for subsequent research. It has to be noted, that the values given in Fig. 5 have been determined for mouse bones. It is known that optimal decalcification times strongly depend on bone size (length and width), but also on the age of the bone donor. In general, larger bones require longer decalcification time and usually, soft bones from infants can be decalcified in shorter times. For large human bone sections, such as the cranial vaults used in an earlier study by Cornelison et al., optimal decalcification times of several days was reported when using EDTA as decalcifying agent^31^. For adjusting decalcification protocols to new bone samples, tracking decalcification status is recommended to choose appropriate decalcification time and avoid too long exposure of the bone to the decalcifying agent. Endpoint of decalcification can be estimated by judging the softness of the tissue, following mass reduction during decalcification, determining the residual Ca^2+^ concentration in the decalcification supernatant or follow radiologically the optical density of the bone in radiographic images^32^.

Decalcification in this study has been performed in at least two different batches employing different mice and independent preparation (decalcification, cutting, staining, and imaging) of bones and evaluated by at least two independent observers. When considering batch-to-batch evaluations and averaging scores from all observers, variations in scores were observed in fluorescence staining images, specifically for the osteoclast parameter. For H&E staining, some variations were noted in the nuclei distribution parameter. In contrast, Raman spectroscopy and imaging showed minimal variations, primarily in the well-resolved features parameter. However, when examining the SNR of the Raman spectra for compact bone and bone marrow, notable variations were observed across batches when samples were treated with EDTA decalcifier and hydrochloric and formic acid (Supplementary Figure S4 a, b). Considering inter-observer variations, the most significant score variation was noticed for the assessment of fluorescence staining images obtained after applying the EDTA decalcification protocol for prolonged time, e.g. 48 h. Subsequently, Raman spectroscopy and imaging after decalcification with formic acid for 24 h, as well as H&E staining after decalcification with 5% nitric acid for 8 h, showed modest variations in assessments. Detailed score values are provided in the Supplementary Evaluation Excel sheet. Due to the discussed impact of the decalcification protocol on the assessment, inconsistency between observers became obvious, particularly when using strong acids. This finding might explain the higher variability in the scoring results obtained with the nitric acid-based decalcification protocol (Fig. 5) and lower scores in Raman spectroscopy and imaging evaluation for hydrochloric and formic acid.

In addition to the variations observed in the evaluation of staining and imaging protocols, it is crucial to discuss other sample preparation artifacts encountered. Our exclusive use of cryosectioning, as opposed to paraffin sectioning, introduced challenges, including structural damage and unintentional loss of bone marrow. These complications were pronounced during H&E and fluorescence staining due to associated intricacies. Occasionally, transfer of the delicate bone slice from well plate, where fluorescence staining was carried out, to the microscope slide led to handling artefacts which affected the overall bone slice integrity. Despite these challenges, we made every effort to obtain the best possible results for our imaging, scoring, and evaluation processes. Since it was the scope of this study to find a suitable decalcification procedure for multimodal imaging including Raman spectroscopy which is seriously hampered by the use of paraffin, we did not consider paraffin sectioning here.

We can conclude that if only H&E staining should be performed for visualization of mouse bone architecture in an ex-vivo context and time is an issue, i.e. short decalcification times are preferred, it is recommended to use 3% nitric acid for 2 h or 8% formic acid for 6 h. For meticulous results, a 48-hour EDTA treatment is advisable. In fluorescence imaging situations, a 48-hour treatment with EDTA is also favorable. For Raman spectroscopy and imaging, a 24-hour treatment with EDTA is recommended. However, if all mentioned imaging methods should be performed on the very same bone sample, a 24-hour decalcification process using EDTA is the preferred option, as it consistently maintains crucial bone features necessary for research. In addition, the type and thickness of the bone must be considered before adopting these suggested decalcification protocols, since we only used mouse bones.

## Materials and methods

### Bone samples, preparation and decalcification

The bones from a total of ten healthy male C57BL/6 mice were used. Mice were bred in-house (animal facility of the Jena University Hospital) and were housed under standardized laboratory conditions. The mice were housed in individually ventilated cages according to institutional guidelines and received food and water ad libitum. Animal husbandry was performed in accordance with the Council Directive of the European Community for the Care and Use of Laboratory Animals (2010/63/EU) and German legislation. Euthanasia of mice was approved by the animal protection authority (TLV, Bad Langensalza, Germany; approval number: TVA 02–040/16), as well as on the legal basis of the notification of the killing of vertebrates for scientific purposes according § 4 (3) of the animal welfare act (10.08.2021). The animals were sacrificed at an age between 13 and 82 weeks by exsanguination in deep anesthesia (100 mg/kg bw ketamine; 10 mg/kg bw xylazine) or cervical dislocation. We complied with the ARRIVE guidelines as much as applicable. The mice were sprayed with 70% ethanol and the abdomen was opened with sterile scissors which was extended to the hind and front legs to remove the fur. Legs were then cut off and surrounding muscle tissue was carefully removed and long bones in particular the Tibia (Ti), Femur (Fe), Humerus (Hu), and Ulna (Ul) were extracted from both the left (L) and right (R) sides of the mice were separated and placed in phosphate buffered saline (PBS).

Directly after isolation of all bones was complete, the bones were immersed in excess amount of 4% formaldehyde (ROTI^®^Histofix, Carl Roth, Karlsruhe Germany) for 18–20 h at a temperature of 4 °C for fixation. Prior to the initiation of the decalcification protocol experiment, the bones were thoroughly cleaned with phosphate buffered saline (PBS, Thermo Fisher Scientific, Waltham, MA, USA).

To comprehensively evaluate various decalcifying agents, this study employed five different decalcifiers which were chosen to represent weak and strong acids as well as chelating agents. Based on a literature review (see Table 1 and Supplementary Table S1), we selected the most commonly used and available decalcifiers from each category. These included strong acids, such as nitric acid in two concentrations (3% and 5%, Carl Roth, Karlsruhe, Germany) and a combination of strong hydrochloric and weak formic acid in the Biodec R formulation from Bio-Optica Milano S.p.A., Milano, Italy. For weak acids, we used 5% trichloroacetic acid as contained in the “Decalcifier Standard” formulation from Carl Roth, Karlsruhe, Germany and 8% formic acid (Sigma-Aldrich/Merck). As a chelating agent, we selected 25% ethylenediamine tetraacetic acid, EDTA as in the “Decalcifier Soft” formulation from Carl Roth. Decalcification durations ranged from 2 to 72 h, with short decalcification times for strong inorganic acids which are known to achieve fast decalcification and longest decalcification times for the chelating agent, known to have slowest decalcification speed (see Table 2). Selected bones were randomly submerged in the decalcifiers, which were changed every 24 h for decalcification periods exceeding 24 h at room temperature, as recommended by manufacturer’s manuals. At specified time points, the bones were thoroughly washed with PBS and submerged in PBS for sectioning.

**Table 2:** Decalcifiers used for the study along with their corresponding time points. Time points were chosen based on available literature information to have short decalcification times for strong acids, longer times for weaker acids and longest times for chelating agents. Additionally, the used protocol abbreviation are introduced.

Decalcifier type	Decalcifier composition	Decalcification times (h)	Abbrevi-ation	Used bones *		
				Batch 1	Batch 2	Batch 3
Strong inorganic acids	3% nitric acid	2	N3_2	Hu	Ti	NA
		6	N3_6	Hu	Ti	Fe
		8	N3_8	Hu	Fe	Ti
	5% nitric acid	2	N5_2	Hu	Ti	NA
		6	N5_6	Hu	Ti	Fe
		8	N5_8	Hu	Fe	Ti
	Hydrochloric and formic acid (Biodec R)	2	HF_2	Ti	Ti	NA
		6	HF_6	Ti	Hu	Fe
		8	HF_8	Ti	Hu	Hu
Weaker organic acids	5% trichloroacetic acid (Decalcifier standard)	12	T_12	Fe	Ti	NA
		24	T_24	Fe	Ti	Hu
		48	T_48	Fe	Hu	Ti
		72	T_72	Fe	Hu	Ul
	8% formic acid	6	F_6	Fe	Hu	NA
		12	F_12	Fe	Hu	Ti
		24	F_24	Fe	Ti	Hu
Chelating agents	25% EDTA (Decalcifier soft)	12	E_12	Ti	Hu	NA
		24	E_24	Ti	Hu	Fe
		48	E_48	Ti	Fe	Hu
		72	E_72	Ti	Fe	Ul

*Tibia (Ti), Femur (Fe), Humerus (Hu), and Ulna (Ul) from mice. NA not available.

Long bones underwent longitudinal dissection (by a scalpel), and were then sectioned transversely using a cryostat (CM1860, Leica Biosystems, Nußloch, Germany) at a temperature of − 20 °C. To do so, bones were directly frozen at − 20 °C on the metal block of the cryostat in PBS as optimized technique for Raman and MALDI imaging^33^. It has to be noted that in our protocol cryosections were used instead of the commonly used paraffin sections in order to be compatible with the Raman analysis protocol, where the paraffin itself would result in a strong Raman signal masking the tissue’s signal of interest. Thus, the protective effect of paraffin on morphology and structure was not utilized here and certain cutting artefacts were observed on the tissue sections, especially if tissue was slightly macerated due to the incubation with the decalcifier. Moreover, we did not use any gel or other commercial freezing preparation since these substances would also interfere with subsequent Raman microspectroscopic analysis. Despite the handling artifacts, we have always considered the best among other tissue sections for imaging and evaluation. For H&E staining, a total of six sections, each measuring 10–12 μm in thickness, were placed on Superfrost microscope slides (Thermo Fisher Scientific). Additionally, three sections measuring 50 μm in thickness were prepared for fluorescence imaging and stored in 96 well plates. Furthermore, two sections of 50 μm thickness were placed on 400 μm quartz glass slides (Nano Quarz Wafer, Langenzenn, Germany) for Raman spectroscopy and imaging purposes. The sample preparation scheme including the cutting is illustrated in Supplemental Figure S7.

### Hematoxylin and eosin staining and imaging

Mayer’s hematoxylin solution and Eosin Y solutions (both, Sigma-Aldrich/Merck, Taufkirchen, Germany) were employed following standard protocols to stain the bone sections, facilitating the visualization of tissue structure. Briefly, tissue sections were hydrated using distilled water. Subsequently, tissue sections were immersed in a hematoxylin solution for 15 min, followed by a gentle rinse with tap water. Sequentially, the sections were exposed to Eosin solution for 2 min, followed by a distilled water rinse. Microscopic examination ensured achievement of the desired staining quality. For dehydration prior to mounting, the sections underwent progressive ethanol (Carl Roth) rinses (70%, 95%, twice 100%), followed by twice rinsing in xylene (Carl Roth). The final step involved embedding the slice using Entellan™ mounting medium (Sigma-Aldrich/Merck).

Bright-field images were captured with a NanoZoomer 2.0-HT slide scanner (Hamamatsu Photonics, Shizuoka, Japan) or using an Axio Observer.Z1 microscope (Carl Zeiss, Jena, Germany), which was equipped with an AxioCam MR R3 camera (Carl Zeiss) and either a Plan Apochromat 40x/0.95 objective or a Plan-Neofluar 63x/1.3 oil immersion objective. In the latter case, the entire sections were scanned in tile-scan mode and subsequently stitched together using Zen 3.3 blue software (Carl Zeiss, version 2.3.69.1017, https://www.micro-shop.zeiss.com/de/de/softwarefinder/software-categories/zen-blue). For viewing, scoring, and analysis purposes, Hamamatsu NDP.view2 (version 2.8.24, https://www.hamamatsu.com/eu/en/product/life-science-and-medical-systems/digital-slide-scanner/U12388-01.html) and Zen 3.1 (blue lite edition, Carl Zeiss, version 3.1, https://www.zeiss.com/microscopy/en/products/software/zeiss-zen-lite.html) software were used, respectively.

### Fluorescence staining and imaging

For immunofluorescence labeling, tissue sections were washed once in PBS, and then subjected to blocking and permeabilization for a minimum of 5 h in a blocking buffer (BB) composed of 2% bovine serum albumin (BSA from Sigma-Aldrich/Merck), 300 mM glycine (Carl Roth, Karlsruhe, Germany), 2% Triton-X100 (Carl Roth), 20% dimethyl sulfoxide (DMSO from Sigma-Aldrich/Merck), and 0.02% sodium azide (Carl Roth) in PBS, containing additionally 5% human serum (Thermo Fisher Scientific). To stain for the osteoblast differentiation marker RUNX2, which is mainly found in pre-osteoblasts, and the osteoclast marker tartrate-resistant acid phosphatase (TRAP), the cryosections (50 μm) were first labelled with the primary antibody, rabbit anti-TRAP in 1:200 (abcam, Cambridge, UK) dilution plus goat anti-RUNX2 (antibodies-online GmbH, Aachen, Germany) 1:200 dilution in BB for 10 days at room temperature while shaking. The slides were washed thrice for 1 h and twice for 10 min in permeabilization buffer (PB) (20% DMSO, 2% Triton X-100, 0.02% sodium azide in PBS). Next, the secondary antibody, DyLight405 donkey anti-rabbit IgG (dianova/BIOZOL, Hamburg, Germany) at 1:200 dilution plus Dylight650 donkey anti-goat IgG (Thermo Fisher Scientific) at dilution 1:250 in BB were applied for 10 days at room temperature while shaking followed by washing thrice for 1 h and twice for 10 min in PB. Subsequently, I555 phalloidin (abnova, Taipei, Taiwan) was used at a concentration of 2 rxn/100 µL to label the cytoskeleton and 0.5 µM SYTOXGreen (Thermo Fisher Scientific) to visualize cell nuclei. Staining was done for 1 day in blocking buffer while shaking. Following staining, the sections were washed three times for 30 min each in PB and once for 10 min in distilled water. Finally, the samples were carefully mounted on glass slides and embedded in VectaShield (Vector Laboratories, Newark, California, US) for subsequent microscopical examination.

Confocal fluorescence images were acquired using a multiphoton confocal laser scanning microscope (LSM 780 META, Carl Zeiss) with Zen 2.2 software (black edition, Carl Zeiss, version 2.2, https://www.micro-shop.zeiss.com/de/de/softwarefinder/software-categories/zen-black/zen-black-system/). Overview images of the whole slides were generated using the “tile scan” option with a Plan Apochromat 20x/0.8 NA objective (Carl Zeiss), on multiple z-planes. Detailed scans were conducted on some regions of selected slides using a 63x/1.15 NA water immersion objective (Carl Zeiss). DyLight405 was excited at 405 nm, and detected in the range of 410–464 nm. SYTOXGreen was excited with a 488 nm light source, spanning a detection range of around 499–597 nm. IF555 phalloidin was excited with a 561 nm light source, with a detection range of 571–633 nm. DyLight650 was excited at 633 nm, with a detection range of 642–695 nm. To prevent channel crosstalk, only DyLight405 and DyLight650 were excited simultaneously, while the other channels were recorded separately. In addition, label-free collagen visualization was achieved by second harmonics generation (SHG) microscopy. This involved using a pulsed Ti: Sa laser (Chameleon, Coherent, Santa Clara, California, US) set to 880 nm, and the backward SHG signal at 437–446 nm was detected.

For manual image viewing and analysis, Zen 3.1 (blue lite edition, Carl Zeiss. version 3.1, https://www.zeiss.com/microscopy/en/products/software/zeiss-zen-lite.html) was used. After image acquisition, the images were manually processed to ensure optimal brightness and contrast levels. This was achieved by adjusting the image parameters to fall within an optimal range, taking into consideration the histogram of each channel. These adjustments were made to enhance visibility and prepare the images for scoring and manual analysis.

### Raman spectroscopy and imaging

Raman spectroscopic characterization of bone tissue sections was performed using an upright confocal Raman microscope (alpha300, WITec, Ulm, Germany) with a 785 nm laser for excitation. The laser power was carefully adjusted to 10 mW at the sample plane to ensure optimal measurement conditions and avoid tissue damage. Back-scattered light was collected through the illumination objective (50x/0.95 NA objective, EC Epiplan from Zeiss). The collected light was then guided to the spectrometer using a multi-mode optical fiber with a diameter of 100 μm. Raman scattered light was diffracted on a 300 g/mm grating, and subsequently recorded on a CCD camera, with the spectral center set to 1999 cm^− 1^, resulting in a spectral window ranging from 400 cm^− 1^ to 3200 cm^− 1^. To ensure accurate calibration, Raman spectra of a silicon wafer and 4-acetamidophenol were recorded prior to the actual measurements. The WITec Control software (version 1.6, available from manufacturer: https://raman.oxinst.com/) software was employed to enable the functionality and control the instrument during the Raman spectroscopic measurements.

An overview bright-field image of the entire tissue slice, measuring 1500 μm x 1500 μm, was obtained using the “Image Stitching” function available within the imaging software and utilizing a 10x/0.3 NA objective (Nikon, Tokio, Japan). To perform in-depth spectral characterization, specific Regions of Interest (ROIs) within the stitched image were selected within compact bone and bone marrow tissue. Square Raman spectroscopic images with sides measuring 20–40 μm were acquired with a 50x/0.95 NA objective (Zeiss). Step size was 0.5 μm and integration time per spectrum was se to two seconds.

### Analysis of Raman data

The Raman image scans of the compact bone were pre-processed and analyzed using R programming language (version 4.1.3, https://cran.r-project.org/bin/windows/base/old/4.1.3/^34^), R Studio software (version 1.4, https://global.rstudio.com/products/rstudio/older-versions/#141717), and hyperSpec^35^and dplyr^36^ packages for data import and manipulation. The pre-processing of the Raman image scans involved the following steps: First, wavenumber correction using the silicon Raman band at 520 cm^− 1^, followed by cosmic ray removal using an automated algorithm^37^. Second, the baseline of the spectra was corrected using the SNIP (Statistics-sensitive Non-linear Iterative Peak-clipping) algorithm^38^ with 400 iterations. A median spectrum per image was calculated after mean normalization, as shown in Fig. 1.

Bone marrow image scans were further analyzed using RamApp (https://ramapp.io/index.html#home). The spectral range was truncated to the regions of 400–1800 cm^− 1^ and 2800–3100 cm^− 1^ and base line was corrected using rubber band method. The N-FINDR algorithm was applied to perform image analysis of the Raman data, leading to the identification and classification of end members for the spectra. This algorithm is known for its effectiveness in identifying spectral components and extracting meaningful information from multi-dimensional datasets.

In addition, the signal-to-noise ratio (SNR) of the spectrum with highest signal intensity was calculated using Python 3.10 programming language^39,40^ to provide an additional score value to assess spectra quality of both compact bone and bone marrow. For this, the following four steps were carried out:

1. Noise Identification and Baseline Correction: The analysis considered noise existing within the spectral range of 1900–2300 cm^− 1^ (silent region). To rectify the spectral baseline, a polynomial function was fitted using the numpy.polynomial package^41^. Calculating the standard deviation of the baseline-corrected array provided a single noise value.
2. Signal Focusing and Smoothing: The Raman band at ~ 1450 cm^− 1^, corresponding to C-H deformation, was identified as the primary band for signal calculation. The spectral range between 1300 and 1600 cm^− 1^ was taken into consideration, and to refine the signal in this region, a Savitzky-Golay filter from the scipy package^42^ was employed for smoothing.
3. Signal Baseline Correction and Value Determination: Baseline correction was carried out using the SNIP algorithm from the pybaselines package^43^ on the spectral range mentioned in step 2, followed by the selection of the maximum value within that range as the signal value.
4. Calculation of final SNR: The signal value derived from step 3, along with the noise value obtained in step 1, was divided to determine the ultimate SNR.

### Scoring

The suitability of the different decalcification methods to obtain high-quality data in the different biophotonic imaging techniques (standard histology, immunofluorescence and Raman microspectroscopy) was evaluated by a scoring system considering several qualitative and additionally some quantitative aspects. For qualitative scoring, two independent observers assessed H&E images, fluorescence images, and the bone marrow portion of Raman images using a predefined scoring scale and established criteria. An overview of the scoring scheme and the criteria is presented in Supplementary Figure S1. Furthermore, the criteria are briefly introduced below for each imaging modality. For each parameter the observers assigned values between 0 and 2 (2 for highest quality and 0 for lowest quality). To ensure accuracy, an additional observer conducted a separate review of the scores assigned by the first two evaluators to identify any significant disparities. If substantial differences were detected (i.e. if the total score of the modality from one batch and condition differed by more than 1), the third evaluator also assessed the images (Supplementary Figure S1d).

For H&E images, qualitative analysis of the staining was performed. We considered two parameters for the compact bone region: cement line visibility and the visibility and staining quality of osteocytes. Three parameters were assessed for the bone marrow region: evaluation of bone marrow nuclei (including distinct cell shapes and staining quality), bone marrow distribution within the section, and cytoplasm visibility across the cells. In a few cases not all parameters could be evaluated, e.g. because the bone marrow was lost during preparation. These parameters were not considered and we choose to average the score per available parameters from all observers followed by averaging this score for all evaluated batches. These average scores per parameter were finally summed up and normalized (see Supplementary Figure S1a).

Immunofluorescence staining after decalcification was evaluated using both qualitative (observer-based) and quantitative measures (see also Supplementary Figure S1b). Qualitative analysis included image parameters for the compact bone and bone marrow regions of the images. Here, scoring based on visual inspection of the compact bone region involved four parameters: staining quality and abundance of (mainly osteocyte) nuclei, staining quality of their cytoskeleton, as well as antibody-based staining of osteoclasts and pre-osteoblasts. The bone marrow region was evaluated based on two parameters: staining of nuclei in bone marrow cells and cytoskeleton staining in bone marrow cells. Similar to the evaluation done for H&E staining, we averaged the score per parameter from all observers followed by averaging this score for all batches and finally summing up all averages of individual parameters and normalizing them to one. Quantitative evaluation of the signal intensity after immunofluorescence staining, included the analysis of the used laser power and detector gain during image acquisition. These parameters were individually adjusted prior to measurement according to the fluorescence signal intensity in a way that the high of the maximum observed signal was around 80–90% of the detector’s dynamic range to achieve best quality images. Low laser power and low detector gain could be used in cases where the staining was of high quality resulting in bright fluorescence signals Therefore, lower laser power and/or detector gain correspond to higher fluorescence signal intensity in the given channel, that imaged osteoclasts, pre-osteoblasts, actin, and nuclei. For each channel we assigned values between 0 and 2 for both laser power and detector gain (e.g. highest values of laser power or detector gain received score 0, while the lowest values were rated by score 2). The final score of set 2 was deduced by summing up all parameters and averaging them between the batches. The total score for fluorescence staining was calculated by averaging the normalized scores of set 1 and set 2 parameters. To obtain a quantitative measure for the signal intensity after immunofluorescence staining, we define a signal intensity index (SII) where now lower values correspond to lower signal intensities. Therefore, we multiplied the used laser power (LP) by the detector gain (DG) and took the inverse value multiplied by 100 for each channel:

$$\:SII=\frac{100}{LP\cdot\:DG}$$

For Raman images, we again evaluated qualitative and quantitative parameters (see also Supplementary Figure S1c). Qualitative analysis focused solely on the obtained bone marrow spectra, as no significant differences were found in the spectra of compact bone. Here, we examined false color images generated after processing the spectra to assess “resolved structures” and identify well-defined characteristics such as distinct shapes of bone marrow cells. Additionally, we analyzed spectra related to these structures in terms of “spectral quality” to identify any present bands associated with bone marrow cells, such as DNA bands, nucleic acid, protein and lipids. Quantitative parameters considered the signal-to-noise ratio of both compact bone and bone marrow spectra. A threshold-based scoring system was derived from the range of SNR values observed in both batches, as described in the subsection “Analysis of Raman data”. For compact bone, due to very minimal variations, scores typically ranged between 1 and 2. The bone marrow section, however, showed greater variability, with scores ranging from 0 to 2. Next, as done for fluorescence analysis, the total score for Raman imaging was calculated by averaging the normalized scores of set 1 and set 2 parameters.

The final score for decalcification considering all imaging modalities was calculated by averaging the normalized scores of H&E staining, fluorescence staining and Raman imaging. The scoring details, along with the corresponding observers and batch-wise scores, are presented in the supplementary evaluation Excel sheet.

## Electronic supplementary material

Below is the link to the electronic supplementary material.

Supplementary Material 1

Supplementary Material 2

## Data Availability

Data is provided within the manuscript and supplementary information files. Further data is available upon request from the corresponding author.
