# Supplementary material for: Qualitative comparison of decalcifiers for mouse bone cryosections for subsequent biophotonic analysis
Source: Sci Rep. 2025 Jan 7;15:1153. doi: 10.1038/s41598-024-84330-2 (PMC11707355; doi:10.1038/s41598-024-84330-2)
Supplement: Supplementary file 1 — Supplementary Material 1 [file 41598_2024_84330_MOESM1_ESM.pdf]

Qualitative Comparison of Decalcifiers for Mouse Bone Cryosections in Subsequent Biophotonic Analysis

Shibarjun Mandal[1], Ramya Motganhalli Ravikumar[1], Astrid Tannert[1,2], Annett Urbanek[1], Rustam R. Guliev[1], Max Naumann[1,3], Sina M. Coldewey[4,5,2], Uta Dahmen[6], Lina Carvalho[7], Luís Bastião Silva[8], Ute Neugebauer[1,2,3*]

1. Leibniz Institute of Photonic Technology (Member of Leibniz Health Technologies, Member of the Leibniz Centre for Photonics in Infection Research, LPI), 07745 Jena, Germany
2. Center for Sepsis Control and Care, Jena University Hospital, 07747 Jena, Germany
3. Institute of Physical Chemistry and Abbe Center of Photonics, Friedrich Schiller University Jena, 07743 Jena, Germany
4. Department of Anesthesiology and Intensive Care Medicine, Jena University Hospital, 07747 Jena, Germany
5. Septomics Research Center, Jena University Hospital, 07745 Jena, Germany
6. Experimental Surgery, Clinic for General, Visceral and Vascular Surgery, Jena University Hospital, 07747 Jena, Germany
7. Institute of Anatomical and Molecular Pathology, Faculty of Medicine, University of Coimbra, 3004-504 Coimbra, Portugal
8. BMD Software, PCI-Creative Science Park, 3830-352 Ílhavo, Portugal

* corresponding author: Ute Neugebauer

Content

Supplementary Figure S1: Schematics of scoring parameters 5
Supplementary Figure S2: Hematoxylin and Eosin stained images 11
Supplementary Figure S3: Fluorescence images 13
Supplementary Figure S4: Average signal intensity index f 14
Supplementary Figure S5: Average Raman signal-to-noise ratios for different decalcifiers. 15
Supplementary Figure S6: Raman spectra and corresponding Raman false colour images 17
Supplementary Figure S7: Bone cutting impression used for tissue preparation and multimodal imaging. 18
Supplementary Table S1: Studies of different decalcifiers 19
Supplementary Table S2: Biomolecular assignments for Raman bands 21

a. Hematoxylin and Eosin Image scoring

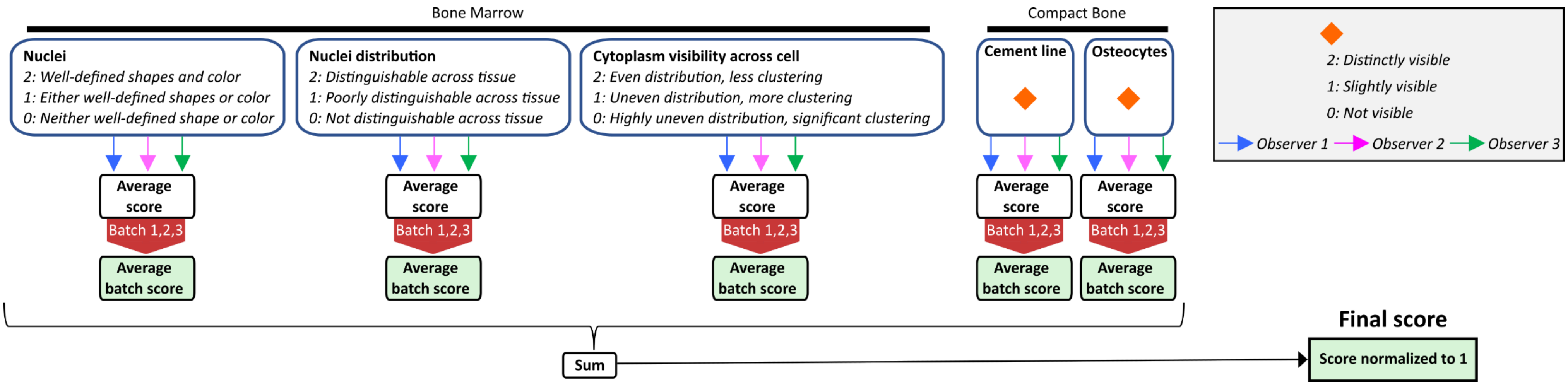

b. Fluorescence Imaging

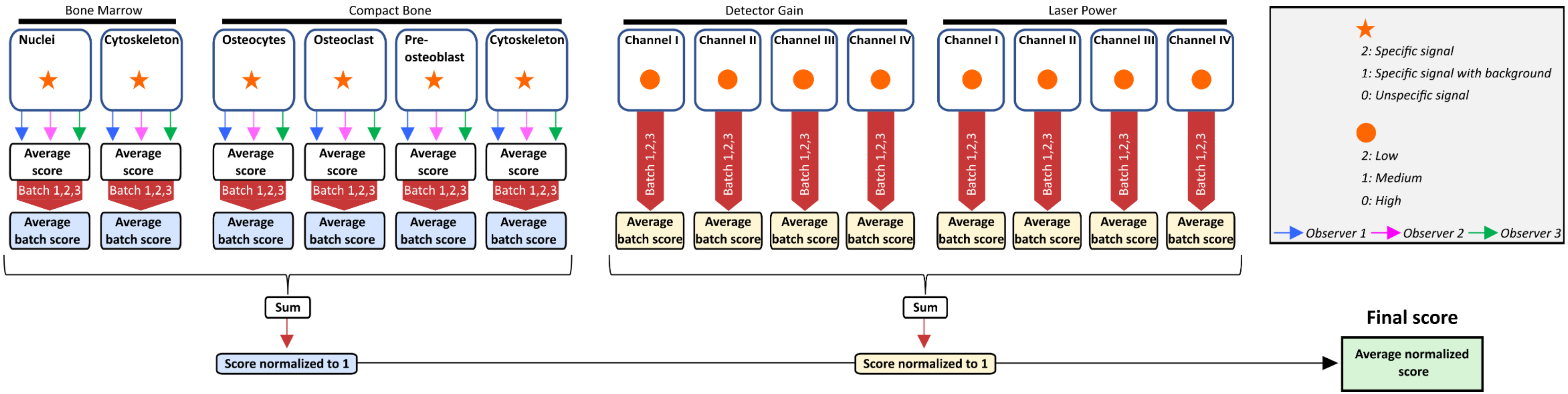

c. Raman Spectroscopy and Imaging

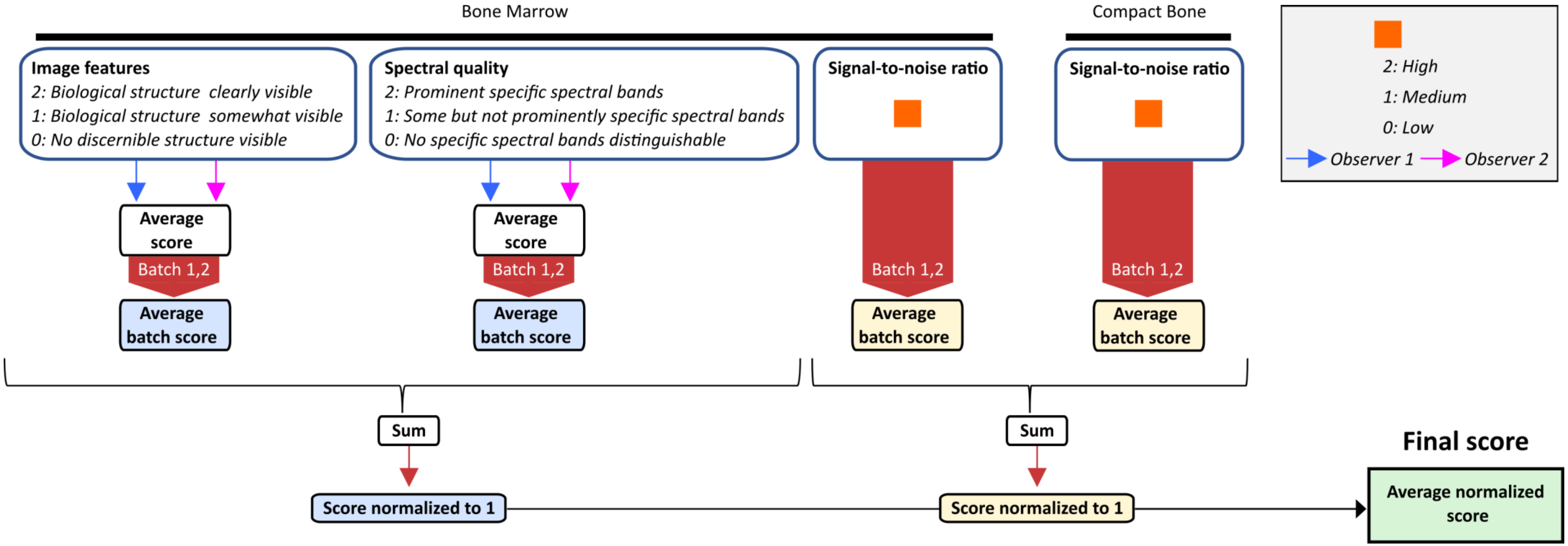

d. Flowchart of scoring process carried out by individual observers

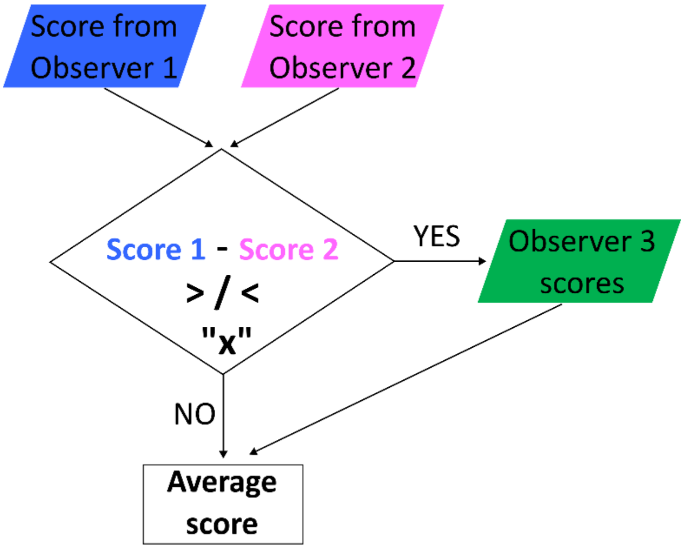

Supplementary Figure S1: Schematics of scoring parameters (a, b, c) Depicts the selection of parameters and scoring methodology applied to images across various imaging modalities and batches. (d) Demonstrates the scoring process carried out by individual observers, presented in the form of a flowchart.

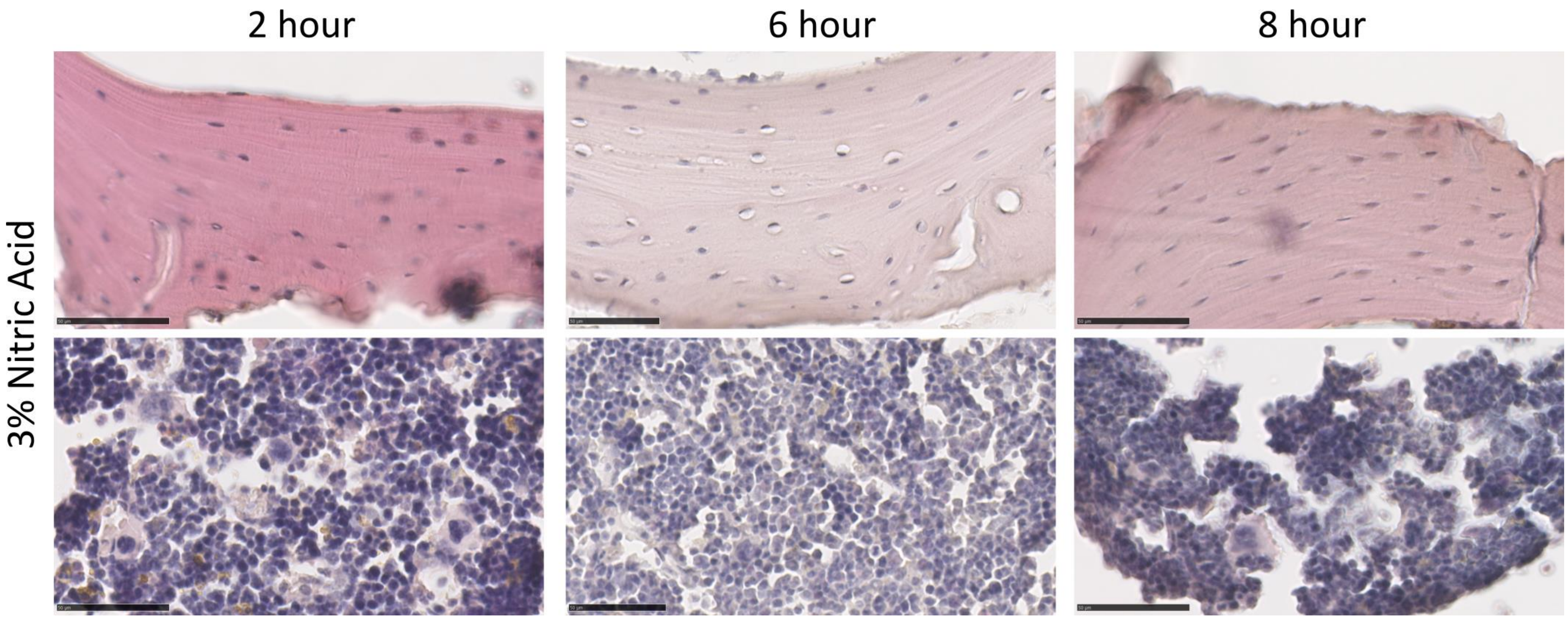
2 hour
6 hour
8 hour
3% Nitric Acid

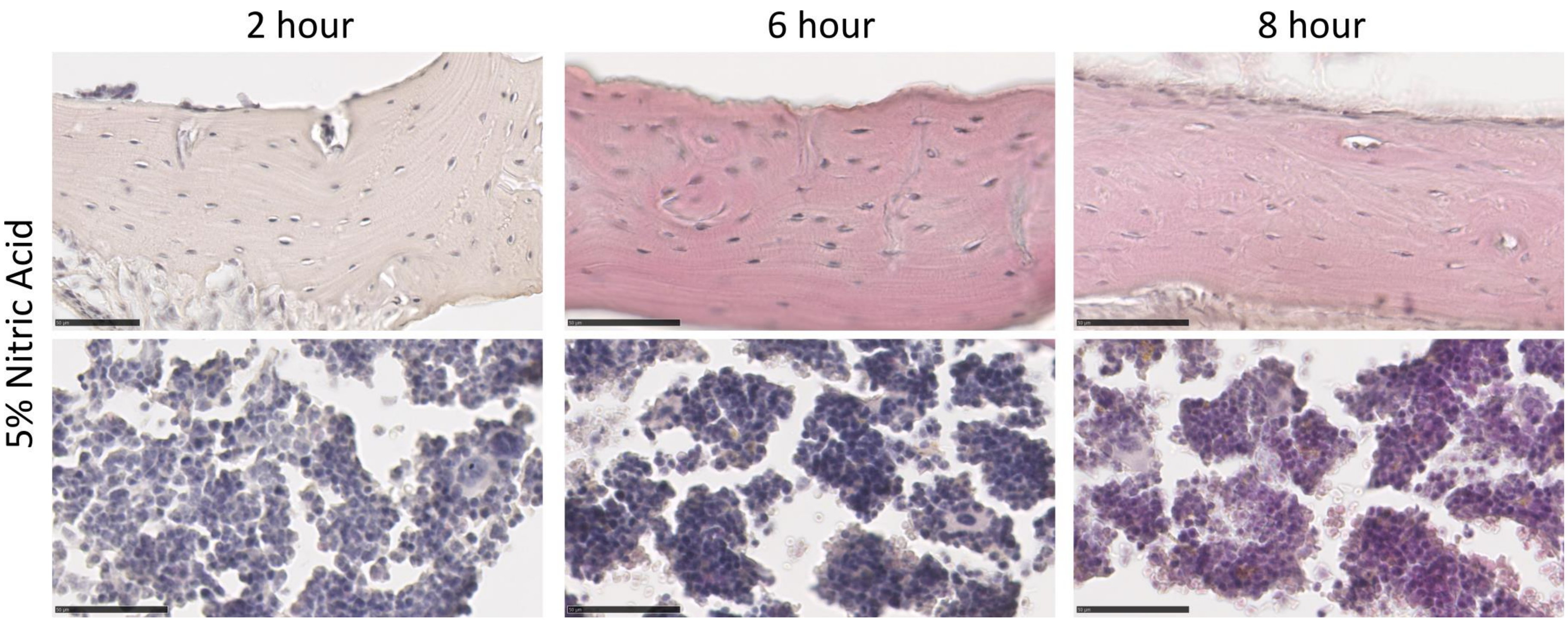
2 hour
6 hour
8 hour
5% Nitric Acid

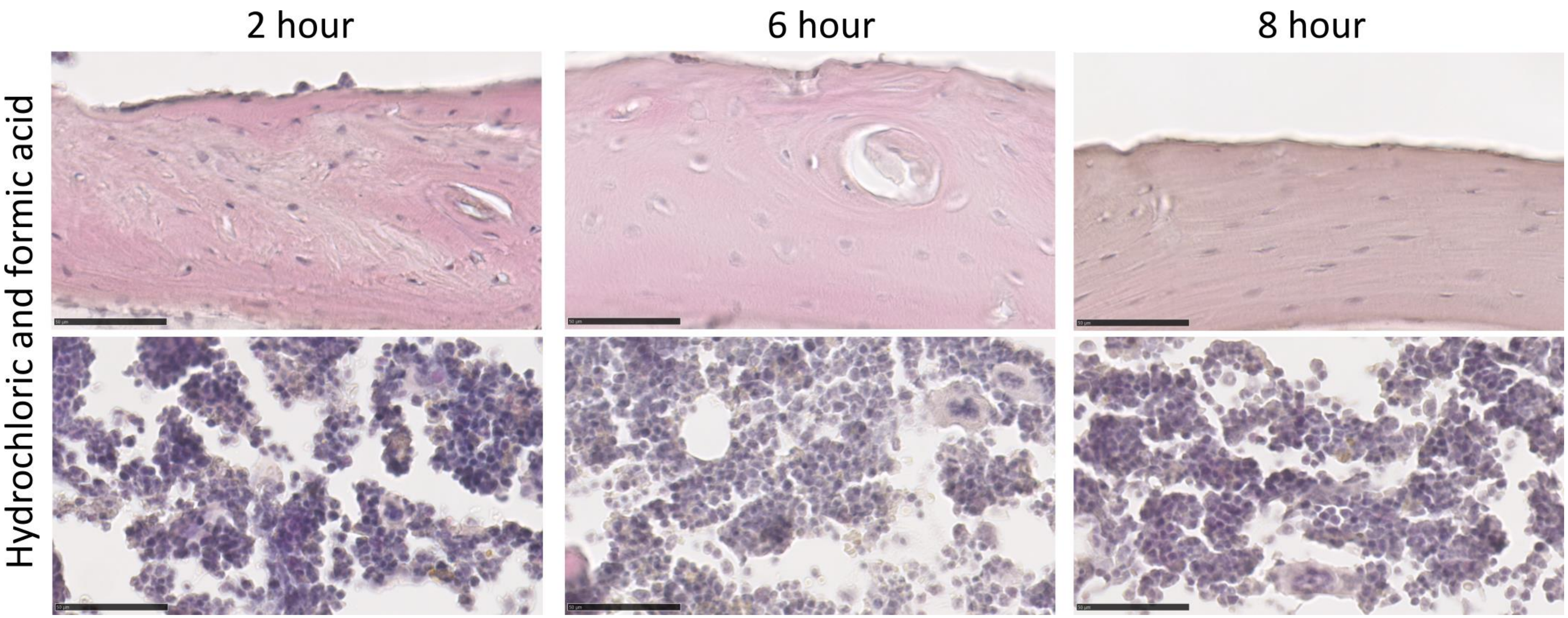
2 hour
6 hour
8 hour
Hydrochloric and formic acid

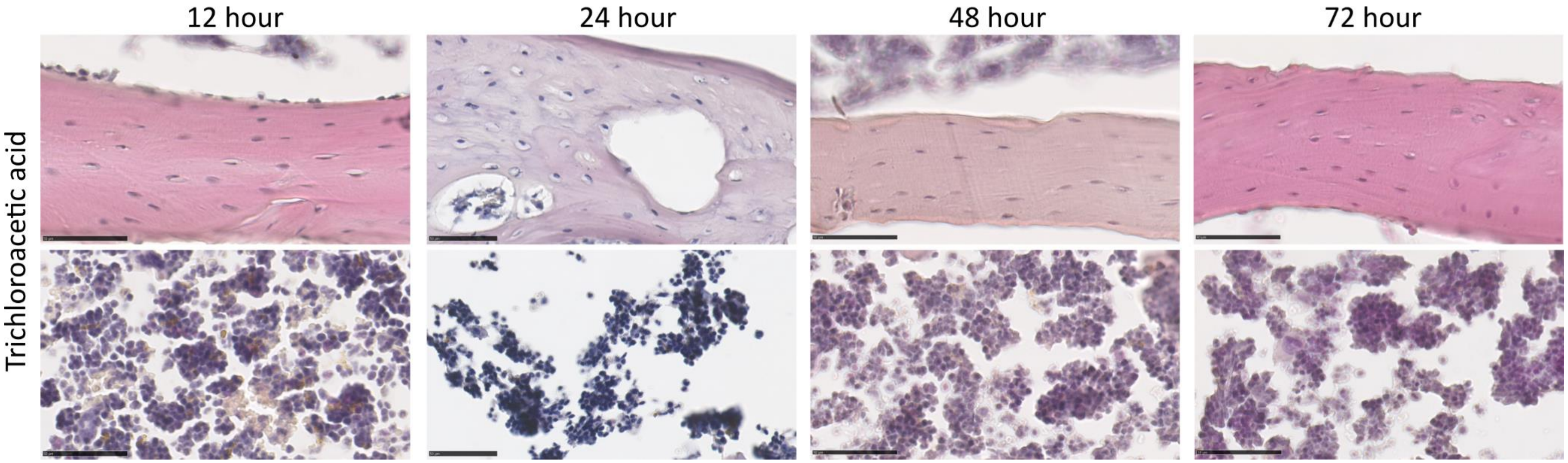
12 hour
24 hour
48 hour
72 hour
Trichloroacetic acid

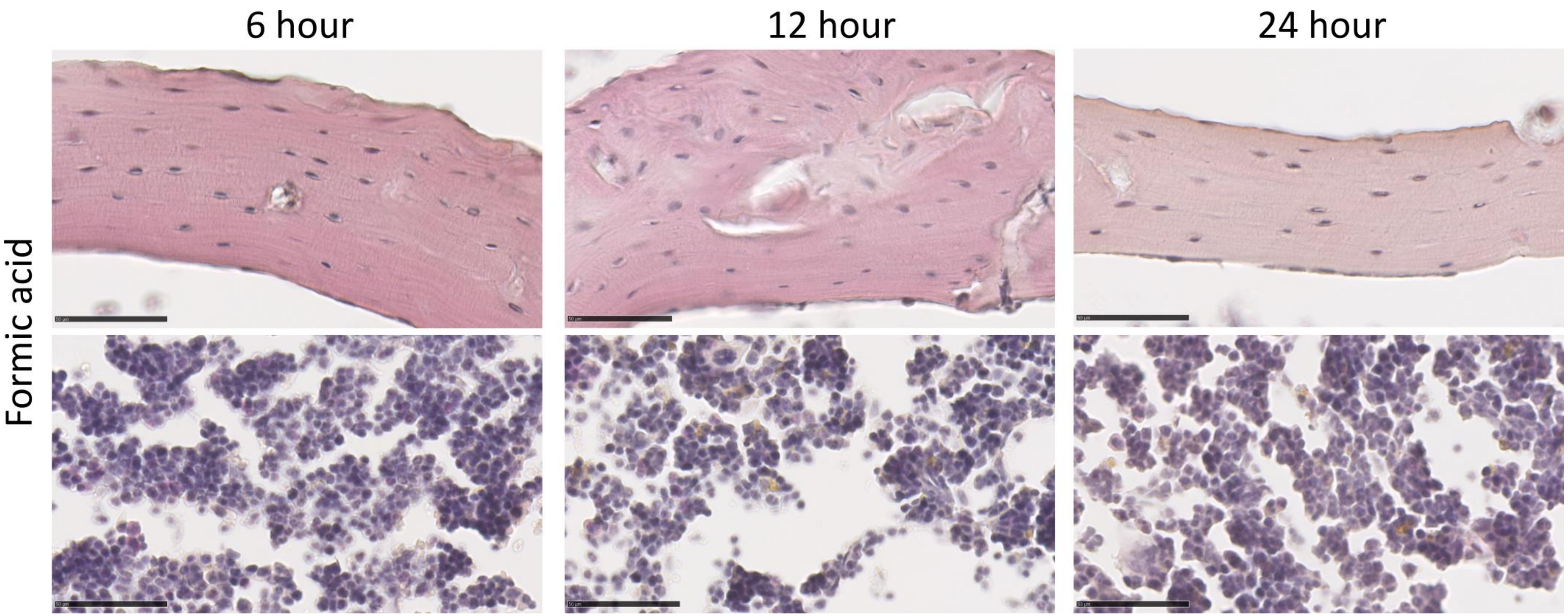
6 hour
12 hour
24 hour
Formic acid

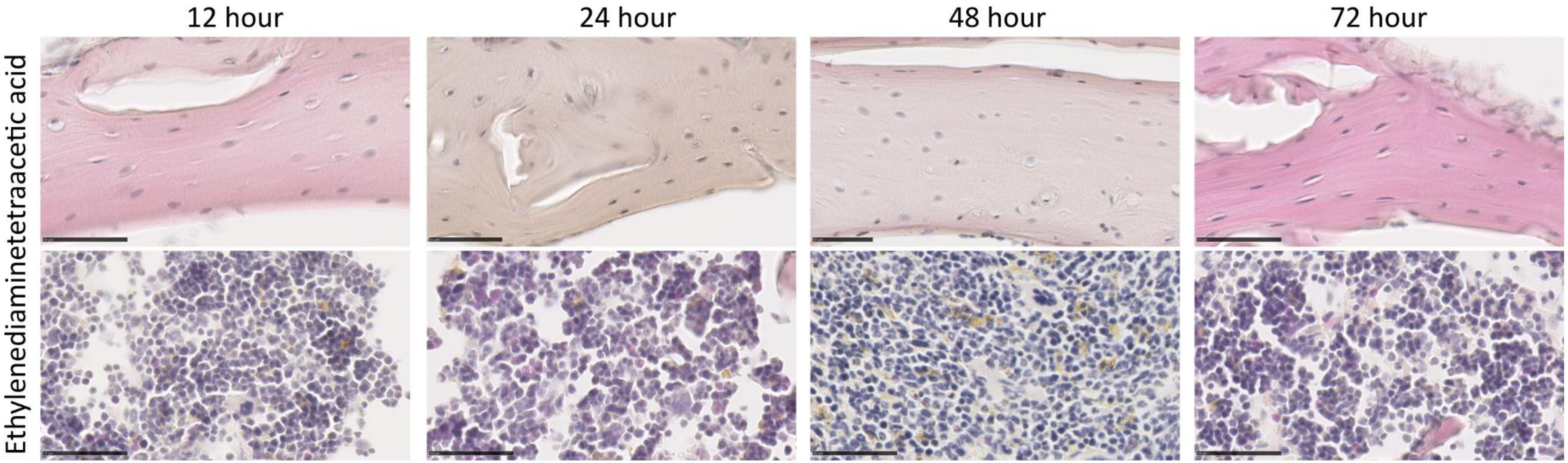

Supplementary Figure S2: Hematoxylin and Eosin stained images (best among the batches) after applying different decalcifying agents for different time points, scale bar: 50 µm

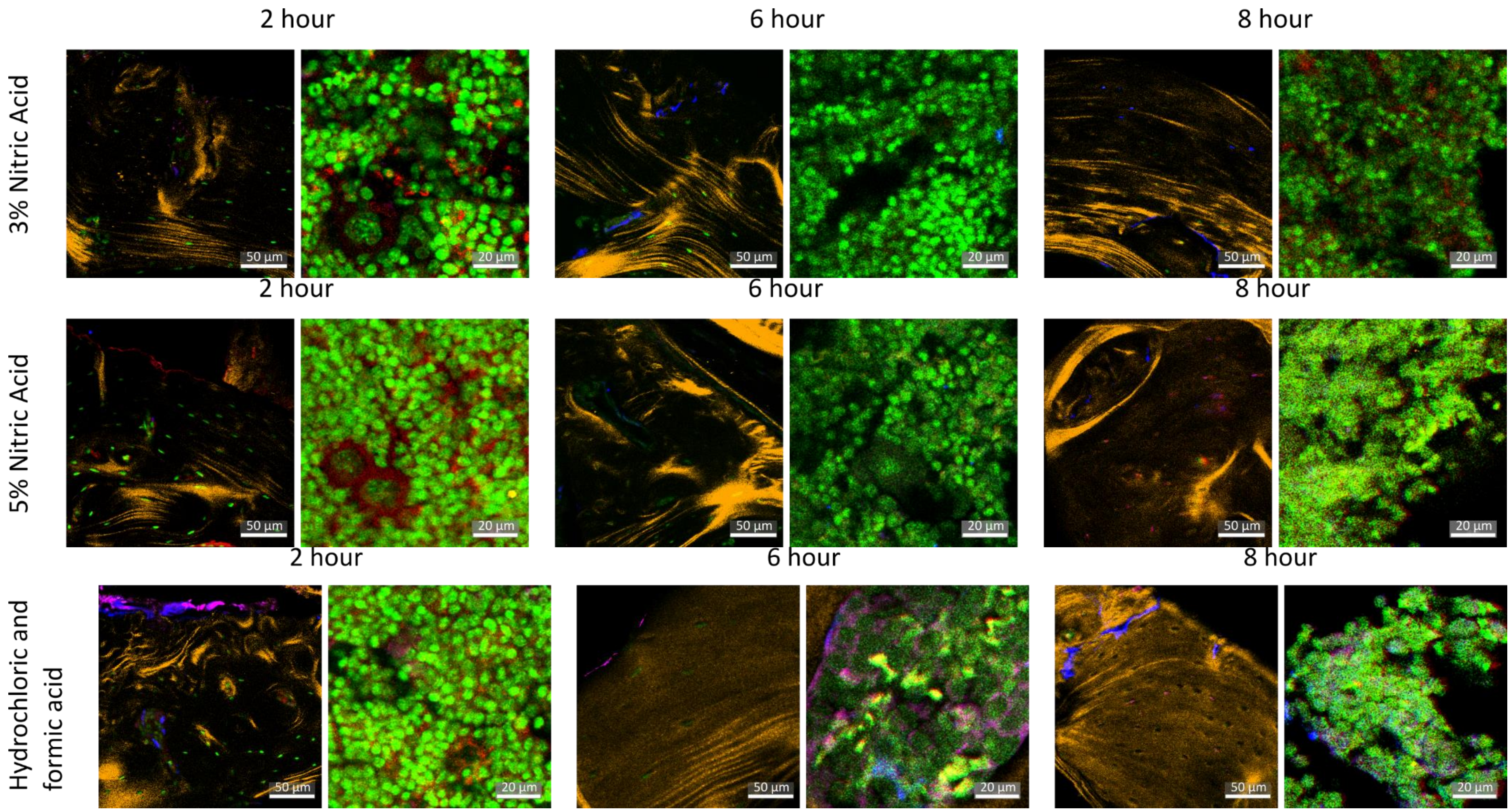
2 hour
6 hour
8 hour
3% Nitric Acid
5% Nitric Acid
Hydrochloric and formic acid
50 µm
20 µm

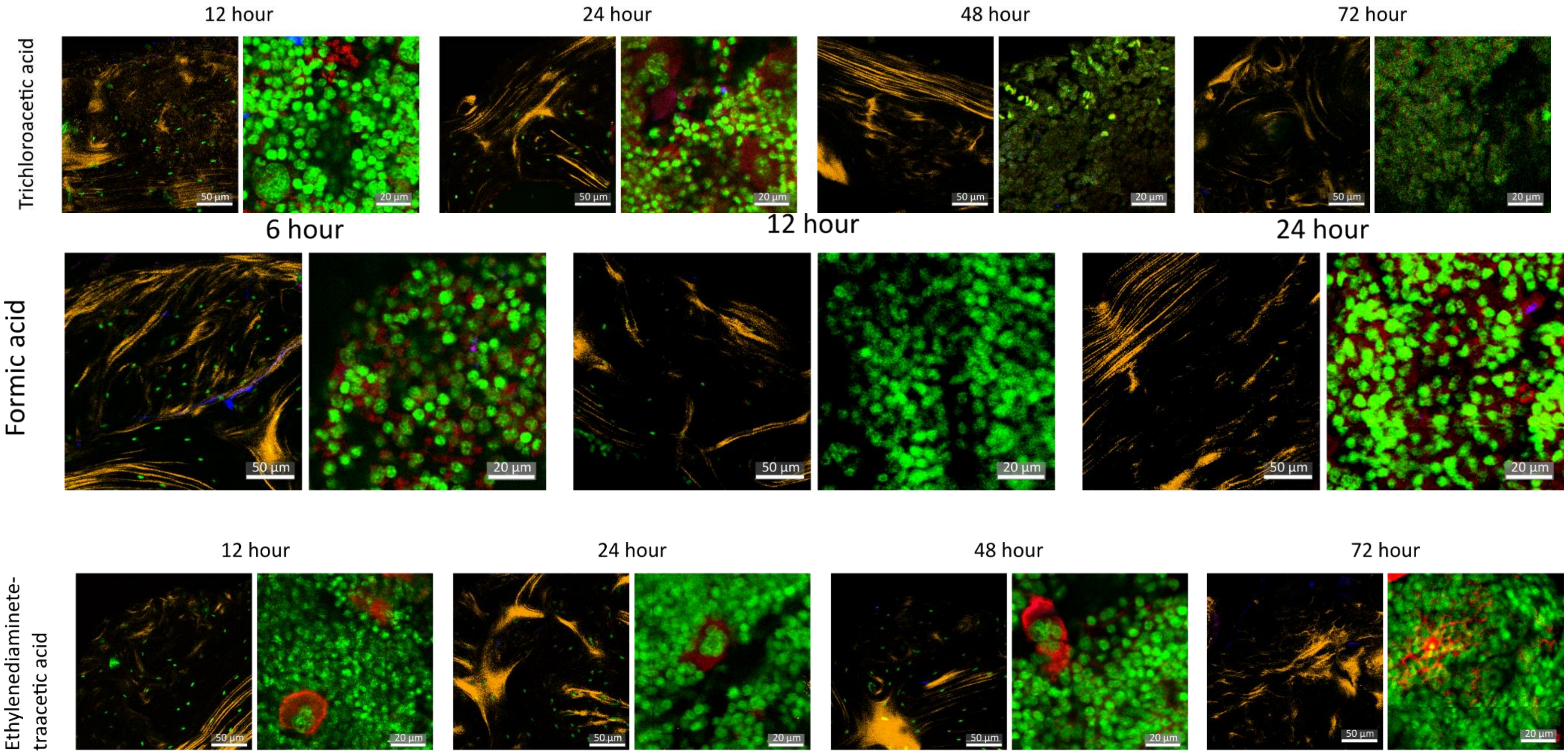

Supplementary Figure S3: Fluorescence images (best among the batches) after applying different decalcifying agents for different time points. Fluorescence staining highlights cell nuclei (green), actin-cytoskeleton (red), collagen lamella (yellow), pre-osteoblasts (pink) and osteoclasts (blue).

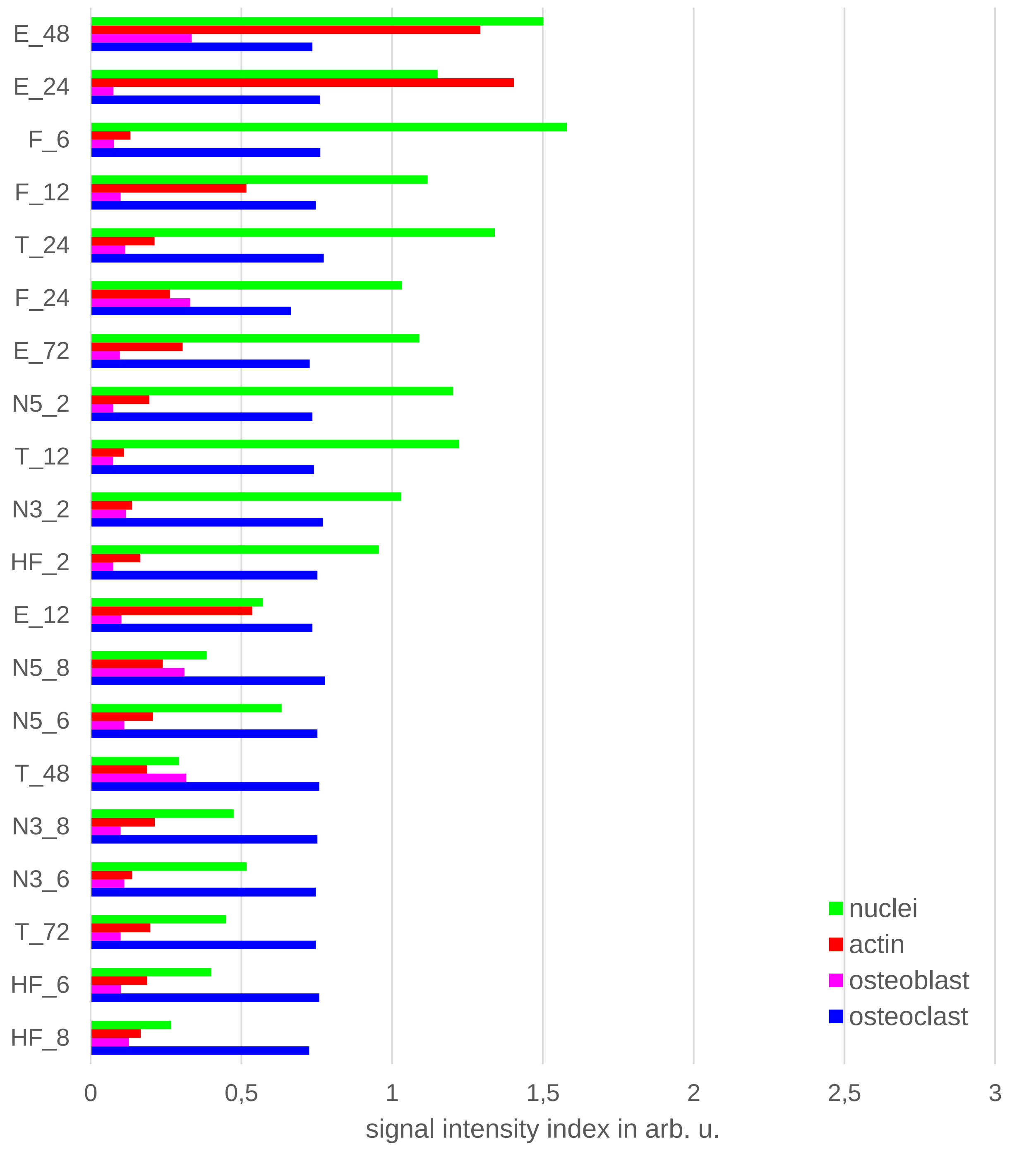

Supplementary Figure S4: Average signal intensity index for the different fluorescence detection channels evaluated after immunofluorescence labelling. Data represent mean±standard deviation of 2-3 batches.

.

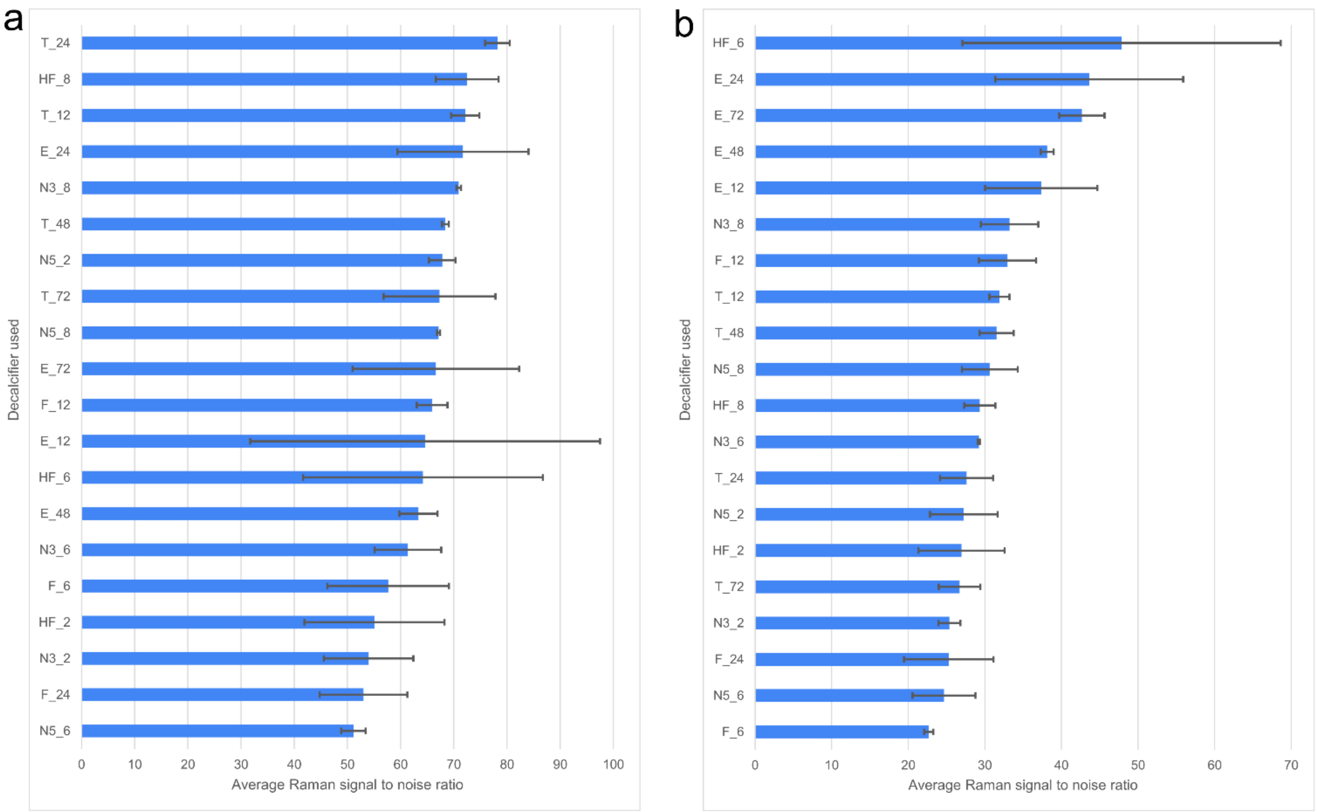

Supplementary Figure S5: Average Raman signal-to-noise ratios for different decalcifiers. (a) SNR values for compact bone (b) SNR values for bone marrow. Error bars represent the standard deviation across different batches, indicating the variability in measurements.

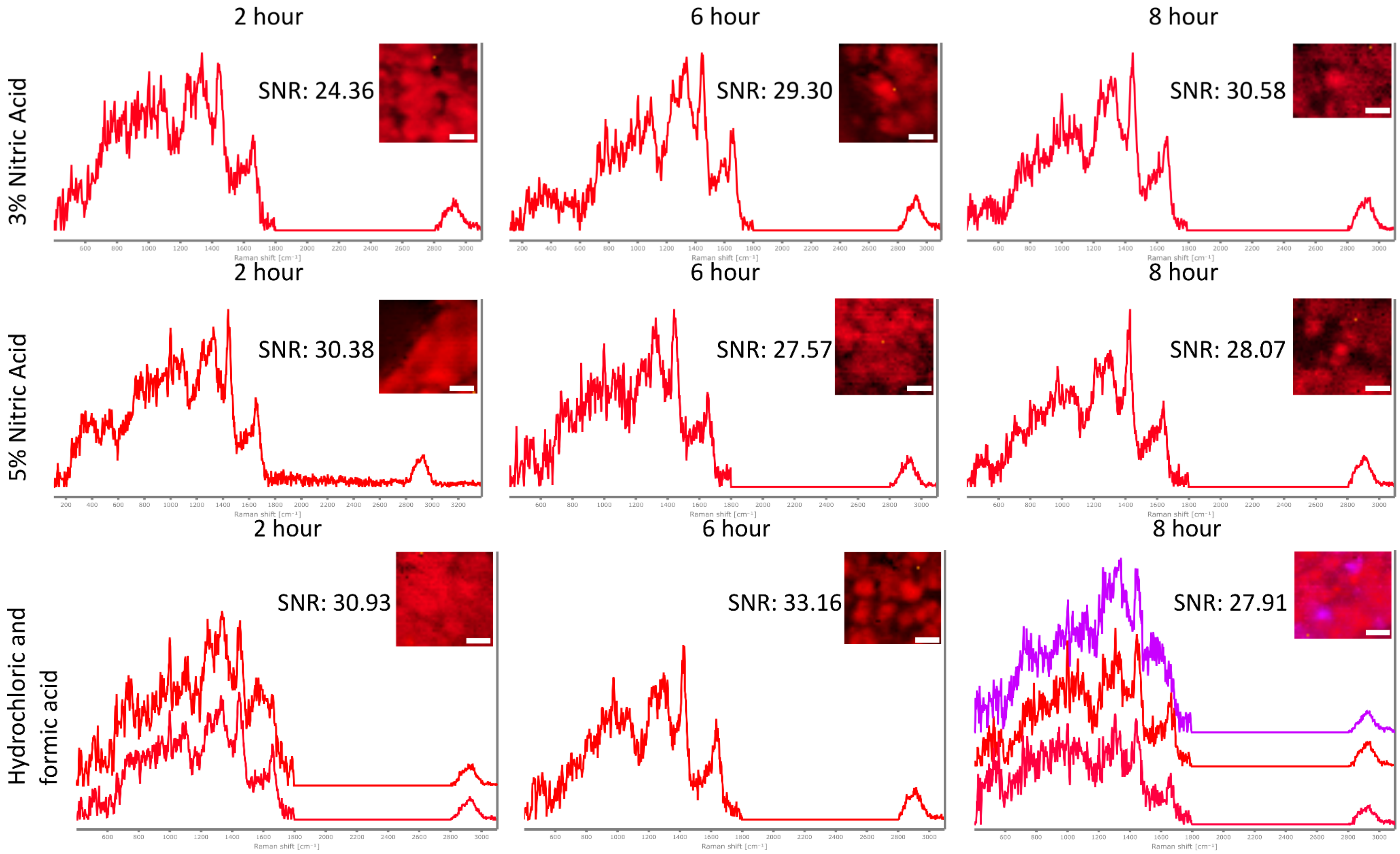
2 hour
6 hour
8 hour
3% Nitric Acid
SNR: 24.36
SNR: 29.30
SNR: 30.58
5% Nitric Acid
SNR: 30.38
SNR: 27.57
SNR: 28.07
Hydrochloric and formic acid
SNR: 30.93
SNR: 33.16
SNR: 27.91

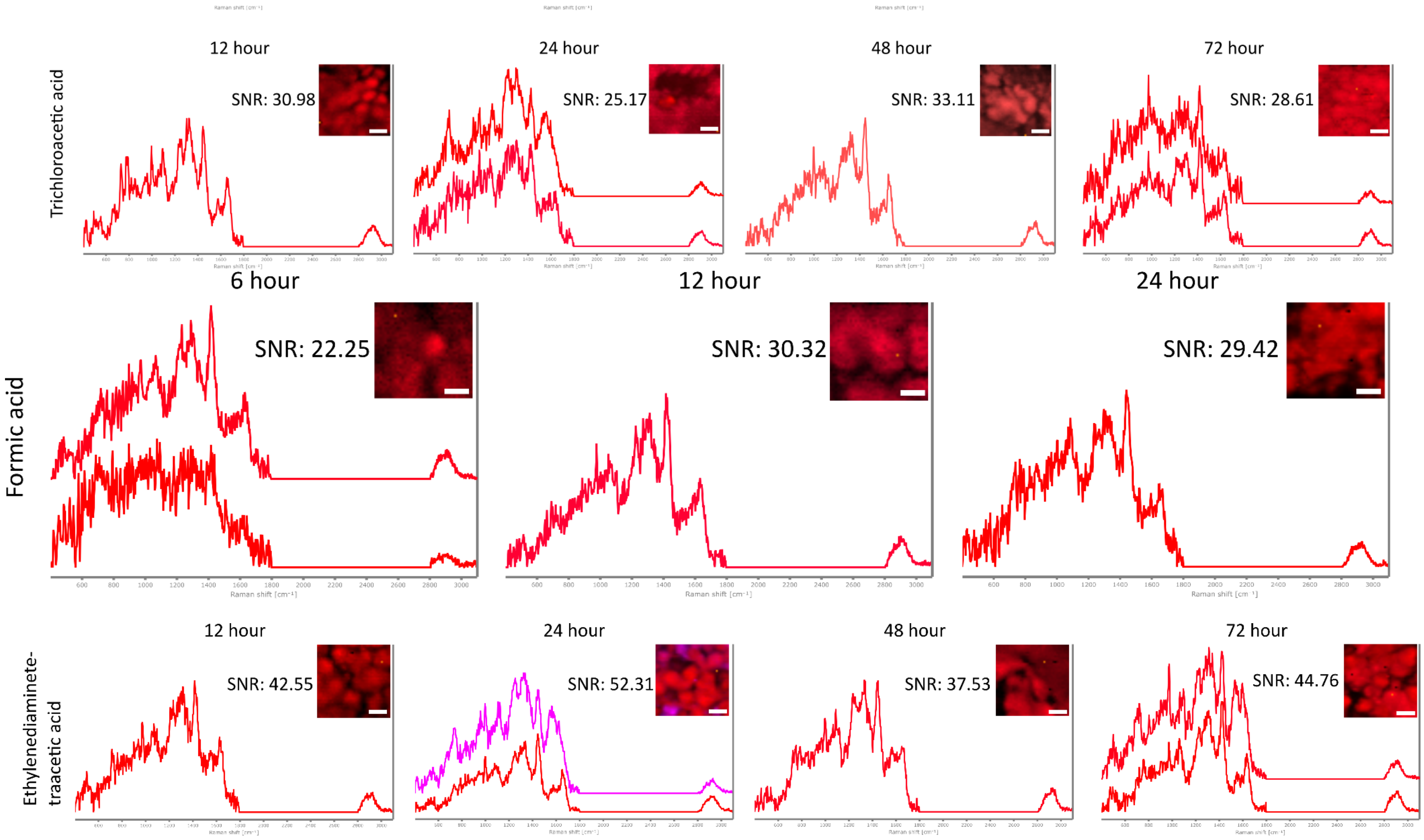

Supplementary Figure S6: Raman spectra and corresponding Raman false colour images (best among the batches) after applying different decalcifying agents for different time points. Scalebar 5 µm

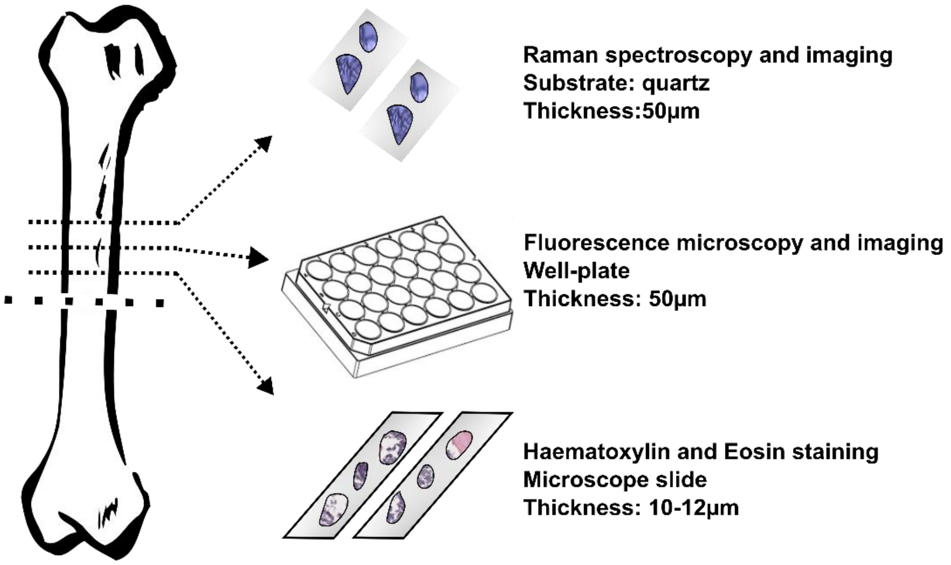

Supplementary Figure S7: Bone cutting impression used for tissue preparation and multimodal imaging. Subsequent sections were used for Raman spectroscopic imaging, immunofluorescence staining and imaging as well as for haematoxylin and eosin (H&E) staining.

Supplementary Table S1: Studies of different decalcifiers published in 2016 and earlier**.** These are categorized by analysis techniques. Studies published 2017 and later are presented in Table 1, main manuscript. Note, only studies using plain decalcifiers are included. Further studies are available that use microwave, ultrasound or heating to improve the decalcification process, however, have not been considered here.

**Reference**	**Bone used**	**Decalcifier used**			**Recommendation for optimal quality in cellular and tissue examination**		
		**Strong acid**	**Weaker organic acid**	**Chelating agents**	**H&E**	**Immunohistochemistry**	**Nucleic acid analysis**
Choi S et al. 2015 [1]	Bone marrow biopsy specimens	N (5%)	F (10%)	EDTA	EDTA	EDTA	EDTA
Jimson et al. 2014 [2]	Human mandible	N (5%)	F (10%)	EDTA	F		
Singh V et al. 2013 [3]	Human bone biopsies	N (5%)	F (10%)	EDTA			EDTA
Brown et al. 2002 [4]	Bone marrow trephine biopsies and autopsy bone samples		F (5% and 10%)				5% F
Walsh et al 1993 [5]	Human femoral heads	N (6%)	F	EDTA			EDTA
Prasad et al. 2013 [6]	Posterior mandible of rat	N (8% and 10%)	F (8% and 10%)	EDTA	F (fast), EDTA (slow)		
Eggert et al. 1979 [7]	Rat maxillae, mandibles, and knee		F (8%)	EDTA	EDTA		

	joints						
Frank J et al. 1993 [8]	Adult rat femur and tibia	N (5%)	F (10%)	EDTA		EDTA	EDTA or F
González-Chávez et al. 2013 [9]	Rat femurs and tibias	N (5%)	F (10%)	EDTA		EDTA or F	
Yamamoto-Fukuda et al. 2000 [10]	Mouse maxilla	N, H	TCA	EDTA			TCA or EDTA

Abbreviations: EDTA: ethylenediamine-tetra acetic acid; N: nitric acid; F: formic acid; H: hydrochloric acid; TCA: trichloroacetic acid

Supplementary Table S2: Biomolecular assignments for Raman bands in bone marrow spectra from decalcified bones

Wavenumber in cm^{-1}	Biomolecular assignment	Reference
~720-788	DNA	[11]
~1003	Ring breathing fro phenylalanine	[11,12]
~1100	RNA	[13]
~1124	Cytochrome c	[13]
~1246-1270	Proteins in form of amide III	[12]
~1310	Saturated lipids	[13]
~1450	C-H deformation, e.g. from protein and lipids	[13], [12]
~1610-1697	Amide I	[11], [14]
~2900-2940	C-H stretching (e.g. from protein and lipids)	[13], [12]

References

1 Choi, S.-E., Hong, S. W. & Yoon, S. O. Proposal of an Appropriate Decalcification Method of Bone Marrow Biopsy Specimens in the Era of Expanding Genetic Molecular Study. *jptm* **49**, 236-242, doi:10.4132/jptm.2015.03.16 (2015).

2 Jimson, S., Masthan, K. M. K. & Elumalai, R. A Comparative Study in Bone Decalcification Using Different Decalcifying Agents. *International Journal of Science and Research* **3**, Paper ID: 02014923 (2014).

3 Singh, V. M. *et al.* Analysis of the effect of various decalcification agents on the quantity and quality of nucleic acid (DNA and RNA) recovered from bone biopsies. *Ann Diagn Pathol* **17**, 322-326, doi:10.1016/j.anndiagpath.2013.02.001 (2013).

4 Brown, R. S. D., Edwards, J., Bartlett, J. W., Jones, C. & Dogan, A. Routine Acid Decalcification of Bone Marrow Samples Can Preserve DNA for FISH and CGH Studies in Metastatic Prostate Cancer. *Journal of Histochemistry & Cytochemistry* **50**, 113-115, doi:10.1177/002215540205000113 (2002).

5 Walsh, L., Freemont, A. J. & Hoyland, J. A. The effect of tissue decalcification on mRNA retention within bone for in-situ hybridization studies. *International journal of experimental pathology* **74**, 237-241 (1993).

6 Prasad, P. & Donoghue, M. A comparative study of various decalcification techniques. *Indian Journal of Dental Research* **28**, 302-308 (2013).

7 Eggert, F. M. & Germain, J. P. Rapid demineralization in acidic buffers. *Histochemistry* **59**, 215-224, doi:10.1007/BF00495669 (1979).

8 Frank, J. D., Balena, R., Masarachia, P., Seedor, J. G. & Cartwright, M. E. The effects of three different demineralization agents on osteopontin localization in adult rat bone using immunohistochemistry. *Histochemistry* **99**, 295-301 (1993).

9 González-Chávez, S. A., Pacheco-Tena, C., Macías-Vázquez, C. E. & Luévano-Flores, E. Assessment of different decalcifying protocols on Osteopontin and Osteocalcin immunostaining in whole bone specimens of arthritis rat model by confocal immunofluorescence. *Int J Clin Exp Pathol* **6**, 1972-1983 (2013).

10 Yamamoto-Fukuda, T. *et al.* Effects of Various Decalcification Protocols on Detection of DNA Strand Breaks by Terminal DUTP Nick End Labelling. *The Histochemical Journal* **32**, 697-702, doi:10.1023/A:1004171517639 (2000).

11 Movasaghi, Z., Rehman, S. & Rehman, I. U. Raman Spectroscopy of Biological Tissues. *Applied Spectroscopy Reviews* **42**, 493-541, doi:10.1080/05704920701551530 (2007).

12 Khalid, M., Bora, T., Ghaithi, A. A., Thukral, S. & Dutta, J. Raman Spectroscopy detects changes in Bone Mineral Quality and Collagen Cross-linkage in Staphylococcus Infected Human Bone. *Scientific Reports* **8**, 9417, doi:10.1038/s41598-018-27752-z (2018).

13 Kukolj, T. *et al.* A Single-Cell Raman Spectroscopy Analysis of Bone Marrow Mesenchymal Stem/Stromal Cells to Identify Inter-Individual Diversity. *International Journal of Molecular Sciences* **23**, 4915 (2022).

14 Morris, M. D. & Mandair, G. S. Raman assessment of bone quality. *Clin Orthop Relat Res* **469**, 2160-2169, doi:10.1007/s11999-010-1692-y (2011).
